# Supplementary material for: Cell Type-Specific TGF-β Mediated EMT in 3D and 2D Models and Its Reversal by TGF-β Receptor Kinase Inhibitor in Ovarian Cancer Cell Lines
Source: Int J Mol Sci. 2019 Jul 22;20(14):3568. doi: 10.3390/ijms20143568 (PMC6678358; doi:10.3390/ijms20143568)
Supplement: Supplementary file 1 [file ijms-20-03568-s001.zip › supplimetary figure 1.pdf]

Supplementory figure 1

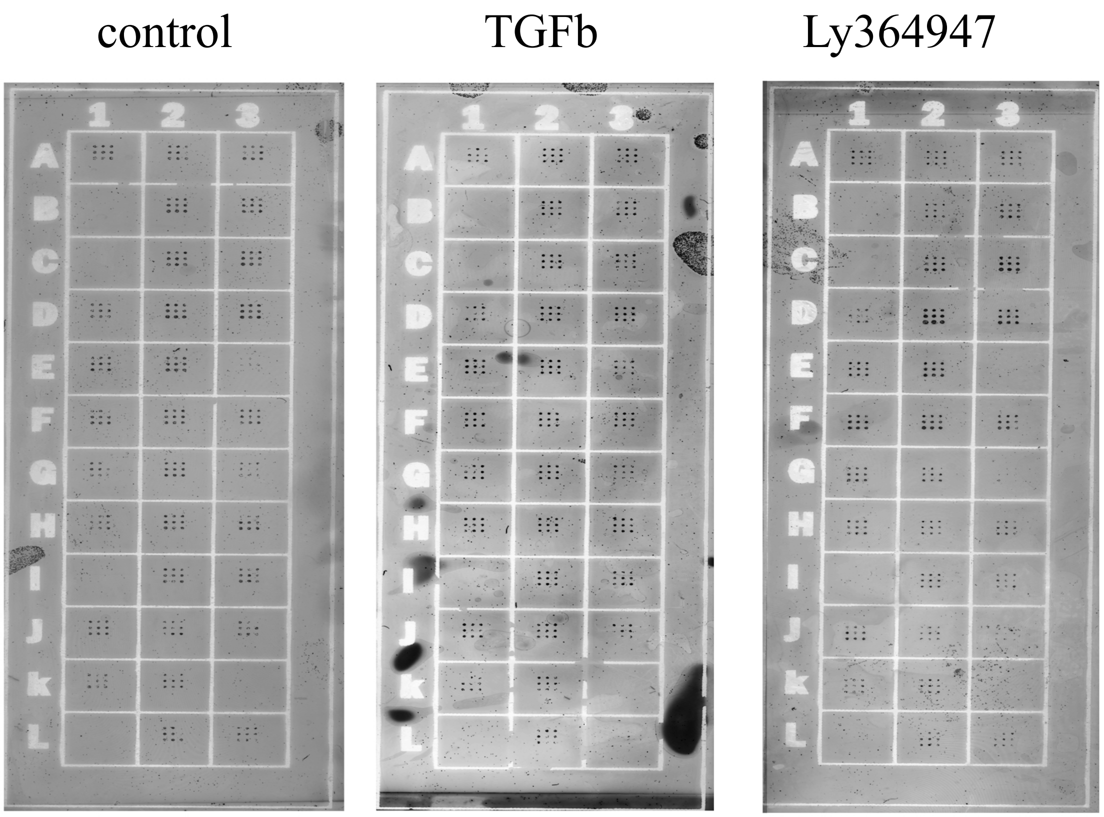

Array Layout

	1	2	3
A	Collagen I (250μg/ml)	Collagen I (125μg/ml) Collagen III (125μg/ml)	Fibronectin (125μg/ml) Collagen IV (125μg/ml)
B	Collagen III (250μg/ml)	Collagen I (125μg/ml) Collagen IV (125μg/ml)	Fibronectin (125μg/ml) Collagen VI (125μg/ml)
C	Collagen IV (250μg/ml)	Collagen I (125μg/ml) Collagen V (125μg/ml)	Fibronectin (125μg/ml) Laminin (125μg/ml)
D	Collagen V (250μg/ml)	Collagen I (125μg/ml) Collagen VI (125μg/ml)	Fibronectin (125μg/ml) Vitronectin(125μg/ml)
E	Collagen VI (250μg/ml)	Collagen I (125μg/ml) Fibronectin (125μg/ml)	Laminin (125μg/ml) Collagen IV (125μg/ml)
F	Fibronectin (250μg/ml)	Collagen I (125μg/ml) Laminin (125μg/ml)	Laminin (125μg/ml) Collagen VI (125μg/ml)
G	Laminin (250μg/ml)	Collagen I (125μg/ml) Vitronectin (125μg/ml)	Vitronectin (125μg/ml) Collagen IV (125μg/ml)
H	Vitronectin (250μg/ml)	Collagen I (125μg/ml) Topoelastin (125μg/ml)	Vitronectin (125μg/ml) Collagen VI (125μg/ml)
I	Tropoelastin (250μg/ml)	Fibronectin (83.3μg/ml) Laminin (83.3μg/ml) Collagen I (83.3μg/ml)	Vitronectin (125μg/ml) Laminin (125μg/ml)
J	Collagen IV (125μg/ml) Collagen VI (125μg/ml)	Fibronectin (83.3μg/ml) Laminin (83.3μg/ml) Collagen IV (83.3μg/ml)	Vitronectin (125μg/ml) Topoelastin (125μg/ml)
K	Collagen III (125μg/ml) Collagen V (125μg/ml)	Vitronectin (62.5μg/ml) Laminin (62.5μg/ml) Collagen I (62.5μg/ml) Collagen IV (62.5μg/ml)	Topoelastin (125μg/ml) Collagen IV (125μg/ml)
L	Negative Control BSA (250μg/ml)	Fibronectin (62.5μg/ml) Laminin (62.5μg/ml) Collagen I (62.5μg/ml) Collagen IV (62.5μg/ml)	Topoelastin (125μg/ml) Collagen VI (125μg/ml)
