# Supplementary material for: Cell Type-Specific TGF-β Mediated EMT in 3D and 2D Models and Its Reversal by TGF-β Receptor Kinase Inhibitor in Ovarian Cancer Cell Lines
Source: Int J Mol Sci. 2019 Jul 22;20(14):3568. doi: 10.3390/ijms20143568 (PMC6678358; doi:10.3390/ijms20143568)
Supplement: Supplementary file 1 [file ijms-20-03568-s001.zip › suppli_table 1.docx]

| **Fold Activation (log2)** | **PA1** | **SKOV3** | **OVCAR3** | **SW626** | **CAOV3** |
| --- | --- | --- | --- | --- | --- |
| EPCAM | 3.738389254 | 0.222628281 | 0.555201232 | 1.634550691 | 0.591436386 |
| OCT | -1.41452539 | 0.331508011 | 2.013752699 | 2.629172087 | 1.986875534 |
| HIF | 1.288842559 | 0.986022949 | 0.580035508 | 0.826325119 | 0.779427826 |
| MYC | -0.269337326 | 0.871306717 | 1.069765091 | 1.014091492 | 1.443744659 |
| CAV | 2.11570549 | -2.622070313 | 0.775948822 | -0.077529907 | 2.28952527 |
| SNAIL | -0.558730423 | 0.48181662 | 0.867202759 | 2.984481812 | 1.778181076 |
| TWIST | 2.76284337 | -1.226268768 | 0.852192581 | 3.070891619 | 0.818198502 |
| MUC | 0.828931153 | -2.578754425 | 2.153484344 | 2.04488945 | 3.959383726 |
| VIM | 1.274195313 | -1.271252275 | 1.325333238 | 1.738594055 | -3.084840059 |
| CD24 | 0.685231507 | 1.066521287 | 1.630719781 | 1.300624847 | 1.303698182 |
| CD44 | -0.768234253 | 0.111271538 | 0.312582642 | -1.267508864 | 3.468816042 |
| CDH2 | 0.963071167 | -0.610940933 | 0.548122406 | 0 | 1.029940963 |
| COL1 | 0.360669464 | 2.599440336 | -0.356484741 | 3.807093382 | 2.638355255 |
| COL3 | 2.190701246 | 10.34201813 | 0.343558639 | 0 | 0 |
| COL4 | 0.954000175 | -0.039617538 | 1.224700332 | -0.851626098 | 3.431987047 |
| COL5 | 0.891773224 | 1.911197662 | 0.533273697 | 2.736856461 | 3.117541075 |
| COL6 | 0.938853562 | 3.132226944 | -1.22402513 | 0 | 3.227620363 |
| MMP2 | 1.680223465 | 0.157706574 | 1.369318366 | 2.565864563 | -1.250200868 |
| FN | 3.490255356 | -0.102362312 | -1.775073051 | -0.393338531 | -0.388947159 |
| FOX | 5.905489445 | -0.48019281 | 0.141775444 | -4.870362759 | 1.235244155 |
| CDH1 | -1.868629456 | -0.147729233 | -0.155950859 | -1.10428369 | -1.72478807 |
| ITGA | 4.552867889 | -0.490844727 | 1.779929757 | -2.290222168 | 0.687124908 |
| IMB | 1.085685134 | -0.102712631 | -0.657568276 | -1.366125703 | -0.767904282 |
| TGFBA | 2.177031755 | -0.235479355 | -1.090661645 | -0.086219788 | -0.854101181 |
| TGFBETA2 | 0 | -0.38160643 | 1.812105536 | -3.274260283 | -1.355266571 |
| DESMO | 3.632069826 | -0.241256073 | 2.011571646 | -1.899904251 | 3.80264163 |
| VITRO | -0.198496506 | -0.242904663 | 1.306180596 | -3.250710726 | -1.481752396 |

Table 1: Gene expression in ovarian cancer cells 3d *vs* 2d
