# Supplementary material for: Cell Type-Specific TGF-β Mediated EMT in 3D and 2D Models and Its Reversal by TGF-β Receptor Kinase Inhibitor in Ovarian Cancer Cell Lines
Source: Int J Mol Sci. 2019 Jul 22;20(14):3568. doi: 10.3390/ijms20143568 (PMC6678358; doi:10.3390/ijms20143568)
Supplement: Supplementary file 1 [file ijms-20-03568-s001.zip › PRIMER LIST 1-suppli file 1.pdf]

GENE NAME	PRIMER NAME
N-cadherin F	AGCCAACCTTAACTGAGGAGT
N-cadherin R	GGCAAGTTGATTGGAGGGATG
COL1A2F	GGCCCTCAAGGTTTCCAAGG
COL1A2R	CACCCTGTGGTCCAACAACTC
COL3A1F	GCCAAATATGTGTCTGTGACTCA
COL3A1R	GGGCGAGTAGGAGCAGTTG
COL5A2F	GACTGTGCCGACCCTGTAAC
COL5A2R	CCTGGACGACCACGTATGC
FN1F	CGGTGGCTGTCAGTCAAAG
FN1R	AAACCTCGGCTTCCTCCATAA
MMP2F	TACAGGATCATTGGCTACACACC
MMP2R	GGTCACATCGCTCCAGACT
VIMF	GACGCCATCAACACCGAGTT
VIMR	CTTTGTCGTTGGTTAGCTGGT
CDH1F	CGAGAGCTACACGTTCACGG
CDH1R	GGGTGTCGAGGGAAAAATAGG
desmoplakinF	GAAGTTTCGCAGACCTGACAT
desmoplakinR	GTATGCACCATTCAACTCCTCG
FOXC2F	CCTCCTGGTATCTCAACCACA
FOXC2R	GAGGGTCGAGTTCTCAATCCC
TGFB2F	CAGCACACTCGATATGGACCA
TGFB2R	CCTCGGGCTCAGGATAGTCT
tgfb1F	GGCCAGATCCTGTCCAAGC
tgfb1R	GTGGGTTTCCACCATTAGCAC
cd44F	CTGCCGCTTTGCAGGTGTA
cd44R	CATTGTGGGCAAGGTGCTATT
itga1F	GTGCTTATTGGTTCTCCGTTAGT
itga1R	CACAAGCCAGAAATCCTCCAT
COL6A2F	TACGGAGAGTGCTACAAGGTG
COL6A2R	GGTCCTGGGAATCCAATGGG
LAMB1F	CACAAGCCCGAACCCTACTG
LAMB1R	GACCACATTTTCAATGAGATGGC
col4a1F	GGACTACCTGGAACAAAAGGG
col4a1R	GCCAAGTATCTCACCTGGATCA
vitronectin 1	GCCTTCACCGACCTCAAGAAC
vitronectin 2	CCCCTGACAGTTGATGCGG
EPCAM1	AATCGTCAATGCCAGTGTACTT
EPCAM2	TCTCATCGCAGTCAGGATCATAA
oct4F	CTTGAATCCCGAATGGAAAGGG

octR	CCTTCCCAAATAGAACCCCCA
beta actinF	CATGTACGTTGCTATCCAGGC
beta actinR	CTCCTTAATGTCACGCACGAT
18SF	GTAACCCGTTGAACCCCATT
18S R.	CCATCCAATCGGTAGTAGCG
HIF1A F	CACCACAGGACAGTACAGGAT
HIF1A R	CGTGCTGAATAATACCACTCACA
MYC F	GTCAAGAGGCGAACACACAAC
MYC R	TTGGACGGACAGGATGTATGC
CAV1F	CATCCCGATGGCACTCATCTG
CAV1 R	TGCACTGAATCTCAATCAGGAAG
snail 1f	ACTGCAACAAGGAATACCTCAG
snail 1 r	GCACTGGTACTTCTTGACATCTG
twist1f	GTCCGCAGTCTTACGAGGAG
twist1 r	GCTTGAGGGTCTGAATCTTGCT
muc16f	CCAGTCCTACATCTTCGGTTGT
muc16r	AGGGTAGTTCCTAGAGGGAGTT
cd24f	CTCCTACCCACGCAGATTTATTC
cd24r	AGAGTGAGACCACGAAGAGAC
gapdh F	GGAGCGAGATCCCTCCAAAAT
gapdh R	GGCTGTTGTCATACTTCTCATGG
