# Supplementary material for: Cell Type-Specific TGF-β Mediated EMT in 3D and 2D Models and Its Reversal by TGF-β Receptor Kinase Inhibitor in Ovarian Cancer Cell Lines
Source: Int J Mol Sci. 2019 Jul 22;20(14):3568. doi: 10.3390/ijms20143568 (PMC6678358; doi:10.3390/ijms20143568)
Supplement: Supplementary file 1 [file ijms-20-03568-s001.zip › Supplimentary figure 2.pdf]

KEGG Pathways for upregulated genes 3D *vs.* 2D

Sublist	Category	Term	RT	Genes	Count	%	P-Value	Benjamini
	KEGG_PATHWAY	Basal cell carcinoma	RT		7	1.9	3.1E-4	5.8E-2
	KEGG_PATHWAY	PI3K-Akt signaling pathway	RT		16	4.3	6.6E-4	6.3E-2
	KEGG_PATHWAY	Pathways in cancer	RT		17	4.6	8.9E-4	5.6E-2
	KEGG_PATHWAY	TGF-beta signaling pathway	RT		7	1.9	2.9E-3	1.3E-1
	KEGG_PATHWAY	Central carbon metabolism in cancer	RT		6	1.6	4.4E-3	1.6E-1
	KEGG_PATHWAY	Hippo signaling pathway	RT		8	2.2	1.4E-2	3.6E-1
	KEGG_PATHWAY	Focal adhesion	RT		9	2.4	2.3E-2	4.8E-1
	KEGG_PATHWAY	HIF-1 signaling pathway	RT		6	1.6	2.5E-2	4.6E-1
	KEGG_PATHWAY	Rap1 signaling pathway	RT		9	2.4	2.6E-2	4.3E-1
	KEGG_PATHWAY	Adipocytokine signaling pathway	RT		5	1.3	3.0E-2	4.5E-1
	KEGG_PATHWAY	Signaling pathways regulating pluripotency of stem cells	RT		7	1.9	3.1E-2	4.3E-1
	KEGG_PATHWAY	Melanoma	RT		5	1.3	3.2E-2	4.1E-1
	KEGG_PATHWAY	Arginine biosynthesis	RT		3	0.8	4.4E-2	4.9E-1
	KEGG_PATHWAY	Proteoglycans in cancer	RT		8	2.2	5.3E-2	5.3E-1
	KEGG_PATHWAY	ECM-receptor interaction	RT		5	1.3	6.0E-2	5.5E-1
	KEGG_PATHWAY	Prostate cancer	RT		5	1.3	6.2E-2	5.4E-1
	KEGG_PATHWAY	HTLV-I infection	RT		9	2.4	6.8E-2	5.5E-1
	KEGG_PATHWAY	Cytokine-cytokine receptor interaction	RT		8	2.2	9.5E-2	6.6E-1
	KEGG_PATHWAY	Mucin type O-Glycan biosynthesis	RT		3	0.8	9.6E-2	6.5E-1
	KEGG_PATHWAY	Pancreatic cancer	RT		4	1.1	9.7E-2	6.3E-1
