# Supplementary material for: Cell Type-Specific TGF-β Mediated EMT in 3D and 2D Models and Its Reversal by TGF-β Receptor Kinase Inhibitor in Ovarian Cancer Cell Lines
Source: Int J Mol Sci. 2019 Jul 22;20(14):3568. doi: 10.3390/ijms20143568 (PMC6678358; doi:10.3390/ijms20143568)
Supplement: Supplementary file 1 [file ijms-20-03568-s001.zip › supplimentary file 2.pdf]

| Gene | logFC |
|---|---|
| ENSG00000140945 | 4.415919297 |
| ENSG00000164125 | 4.065567112 |
| ENSG00000197550 | 2.979478467 |
| ENSG00000139329 | 2.967894714 |
| ENSG00000075275 | 2.833340504 |
| ENSG00000198842 | 2.801643685 |
| ENSG00000175040 | 2.577334931 |
| ENSG00000003436 | 2.545252042 |
| ENSG00000145431 | 2.542358697 |
| ENSG00000148848 | 2.540973427 |
| ENSG00000047457 | 2.536595129 |
| ENSG00000100867 | 2.505787083 |
| ENSG00000184828 | 2.504372838 |
| ENSG00000174718 | 2.497170656 |
| ENSG00000183778 | 2.47860181 |
| ENSG00000197301 | 2.394451772 |
| ENSG00000107249 | 2.379363034 |
| ENSG00000120075 | 2.337574452 |
| ENSG00000172927 | 2.301554582 |
| ENSG00000142623 | 2.262921131 |
| ENSG00000267942 | 2.259538443 |
| ENSG00000166257 | 2.242218555 |
| ENSG00000118785 | 2.212876826 |
| ENSG00000141736 | 2.204272604 |
| ENSG00000183873 | 2.192124257 |
| ENSG00000164161 | 2.161281519 |
| ENSG00000113924 | 2.151961076 |
| ENSG00000166415 | 2.1428262 |
| ENSG00000172164 | 2.117446526 |
| ENSG00000196562 | 2.116575964 |
| ENSG00000095739 | 2.114854921 |
| ENSG00000078114 | 2.094976794 |
| ENSG00000154856 | 2.094225456 |
| ENSG00000149328 | 2.092600318 |
| ENSG00000136167 | 2.0799331 |
| ENSG00000168646 | 2.071136347 |
| ENSG00000163032 | 2.046589422 |
| ENSG00000121690 | 2.029338972 |
| ENSG00000048052 | 2.00395821 |

| | |
|---|---|
| ENSG00000166750 | 1.993026011 |
| ENSG00000187889 | 1.990162568 |
| ENSG00000067715 | 1.962892431 |
| ENSG00000131969 | 1.951830228 |
| ENSG00000268621 | 1.917678819 |
| ENSG00000169908 | 1.917292813 |
| ENSG00000125845 | 1.898968672 |
| ENSG00000197635 | 1.897862172 |
| ENSG00000064300 | 1.891851131 |
| ENSG00000188707 | 1.856464001 |
| ENSG00000140807 | 1.848115952 |
| ENSG00000244242 | 1.845586408 |
| ENSG00000163435 | 1.843043003 |
| ENSG00000136542 | 1.839727587 |
| ENSG00000099139 | 1.835832463 |
| ENSG00000189223 | 1.819287572 |
| ENSG00000064787 | 1.816880776 |
| ENSG00000253910 | 1.810199589 |
| ENSG00000136383 | 1.806063082 |
| ENSG00000114115 | 1.803343749 |
| ENSG00000151632 | 1.792422009 |
| ENSG00000178752 | 1.788235431 |
| ENSG00000184613 | 1.752562389 |
| ENSG00000052795 | 1.748150502 |
| ENSG00000113248 | 1.736863463 |
| ENSG00000116990 | 1.729510588 |
| ENSG00000108511 | 1.722318963 |
| ENSG00000261685 | 1.721744668 |
| ENSG00000196757 | 1.720136799 |
| ENSG00000197857 | 1.717506469 |
| ENSG00000160229 | 1.714192071 |
| ENSG00000119547 | 1.713290044 |
| ENSG00000046889 | 1.710920522 |
| ENSG00000151468 | 1.70505589 |
| ENSG00000198796 | 1.704654601 |
| ENSG00000138639 | 1.691995943 |
| ENSG00000164114 | 1.689986703 |
| ENSG00000253846 | 1.683917599 |
| ENSG00000152492 | 1.681826107 |
| ENSG00000175600 | 1.673970533 |

| | |
|---|---|
| ENSG00000135925 | 1.667986424 |
| ENSG00000118997 | 1.66581528 |
| ENSG00000102547 | 1.664163388 |
| ENSG00000162849 | 1.663906097 |
| ENSG00000135218 | 1.661612871 |
| ENSG00000125384 | 1.660212626 |
| ENSG00000198948 | 1.657971357 |
| ENSG00000128849 | 1.646524109 |
| ENSG00000067798 | 1.645076178 |
| ENSG00000173432 | 1.642938891 |
| ENSG00000127399 | 1.641038007 |
| ENSG00000196268 | 1.631487856 |
| ENSG00000109756 | 1.623444938 |
| ENSG00000269825 | 1.622332832 |
| ENSG00000139687 | 1.622323433 |
| ENSG00000088992 | 1.621638501 |
| ENSG00000163536 | 1.621478719 |
| ENSG00000125378 | 1.61980706 |
| ENSG00000169435 | 1.616972983 |
| ENSG00000125730 | 1.614749352 |
| ENSG00000106003 | 1.611050984 |
| ENSG00000100393 | 1.607001015 |
| ENSG00000261087 | 1.605250412 |
| ENSG00000107159 | 1.604144601 |
| ENSG00000102760 | 1.597648596 |
| ENSG00000171497 | 1.594391674 |
| ENSG00000187624 | 1.588590149 |
| ENSG00000197415 | 1.586823072 |
| ENSG00000168386 | 1.582921026 |
| ENSG00000120093 | 1.579802887 |
| ENSG00000171346 | 1.577299571 |
| ENSG00000164124 | 1.576538841 |
| ENSG00000111885 | 1.574616765 |
| ENSG00000156463 | 1.561920318 |
| ENSG00000090530 | 1.561909962 |
| ENSG00000213976 | 1.560277063 |
| ENSG00000158258 | 1.551263378 |
| ENSG00000188277 | 1.535973886 |
| ENSG00000123689 | 1.532352023 |
| ENSG00000134627 | 1.517718893 |

| | |
|---|---|
| ENSG00000171503 | 1.51309893 |
| ENSG00000170266 | 1.506852324 |
| ENSG00000248449 | 1.504462432 |
| ENSG00000002587 | 1.497870576 |
| ENSG00000109610 | 1.497783079 |
| ENSG00000167766 | 1.495689508 |
| ENSG00000058335 | 1.488946404 |
| ENSG00000250072 | 1.485066937 |
| ENSG00000105281 | 1.478237466 |
| ENSG00000184226 | 1.470488402 |
| ENSG00000119138 | 1.470219617 |
| ENSG00000156804 | 1.469592619 |
| ENSG00000240668 | 1.466943805 |
| ENSG00000182795 | 1.466611694 |
| ENSG00000188001 | 1.463175683 |
| ENSG00000176945 | 1.462833322 |
| ENSG00000185674 | 1.458351669 |
| ENSG00000074416 | 1.456570818 |
| ENSG00000183779 | 1.449471575 |
| ENSG00000153551 | 1.43793314 |
| ENSG00000137502 | 1.424846411 |
| ENSG00000188549 | 1.419085411 |
| ENSG00000078814 | 1.418644999 |
| ENSG00000179750 | 1.417608847 |
| ENSG00000110723 | 1.413189579 |
| ENSG00000135362 | 1.41158002 |
| ENSG00000164308 | 1.404511372 |
| ENSG00000113657 | 1.403636878 |
| ENSG00000267934 | 1.403391611 |
| ENSG00000230606 | 1.40280537 |
| ENSG00000133067 | 1.400983921 |
| ENSG00000128683 | 1.400683531 |
| ENSG00000188167 | 1.39325778 |
| ENSG00000119042 | 1.393225579 |
| ENSG00000109654 | 1.387736347 |
| ENSG00000240476 | 1.387644431 |
| ENSG00000101276 | 1.385889069 |
| ENSG00000128536 | 1.38500264 |
| ENSG00000267010 | 1.383797023 |
| ENSG00000272341 | 1.377443373 |

| | |
|---|---|
| ENSG00000156427 | 1.373610216 |
| ENSG00000075213 | 1.372073782 |
| ENSG00000138131 | 1.369153585 |
| ENSG00000154027 | 1.366053266 |
| ENSG00000172175 | 1.359073169 |
| ENSG00000248100 | 1.358956331 |
| ENSG00000126005 | 1.357535376 |
| ENSG00000104983 | 1.355762158 |
| ENSG00000204335 | 1.354764446 |
| ENSG00000246898 | 1.351655553 |
| ENSG00000078269 | 1.348978528 |
| ENSG00000251493 | 1.343750725 |
| ENSG00000115419 | 1.342913666 |
| ENSG00000122641 | 1.342593092 |
| ENSG00000161395 | 1.33924194 |
| ENSG00000168675 | 1.336749706 |
| ENSG00000260231 | 1.336549164 |
| ENSG00000272068 | 1.329419568 |
| ENSG00000116678 | 1.326631117 |
| ENSG00000124920 | 1.323499351 |
| ENSG00000170421 | 1.322818282 |
| ENSG00000155792 | 1.321382002 |
| ENSG00000165025 | 1.32016661 |
| ENSG00000109762 | 1.319426699 |
| ENSG00000123095 | 1.316258367 |
| ENSG00000164379 | 1.311229455 |
| ENSG00000144366 | 1.310708462 |
| ENSG00000254285 | 1.308449694 |
| ENSG00000224520 | 1.306176897 |
| ENSG00000083857 | 1.300951001 |
| ENSG00000088970 | 1.298004427 |
| ENSG00000101224 | 1.286623824 |
| ENSG00000152409 | 1.285978294 |
| ENSG00000087253 | 1.283908652 |
| ENSG00000163281 | 1.282242009 |
| ENSG00000153292 | 1.278774219 |
| ENSG00000185920 | 1.277659124 |
| ENSG00000141741 | 1.274488447 |
| ENSG00000250539 | 1.273764316 |
| ENSG00000077063 | 1.271409558 |

| | |
|---|---|
| ENSG00000108375 | 1.26798104 |
| ENSG00000186081 | 1.266891504 |
| ENSG00000176092 | 1.264749412 |
| ENSG00000120149 | 1.260006149 |
| ENSG00000120327 | 1.259475915 |
| ENSG00000116117 | 1.258388236 |
| ENSG00000163328 | 1.258345688 |
| ENSG00000205208 | 1.256149691 |
| ENSG00000154319 | 1.249151701 |
| ENSG00000112394 | 1.247842419 |
| ENSG00000136869 | 1.240068894 |
| ENSG00000125966 | 1.236459708 |
| ENSG00000083814 | 1.233172699 |
| ENSG00000171291 | 1.22873446 |
| ENSG00000175538 | 1.226100107 |
| ENSG00000237973 | 1.22544986 |
| ENSG00000171295 | 1.223980488 |
| ENSG00000131748 | 1.22383424 |
| ENSG00000214922 | 1.222687814 |
| ENSG00000214417 | 1.221552983 |
| ENSG00000181016 | 1.221148501 |
| ENSG00000130045 | 1.218880137 |
| ENSG00000231203 | 1.218378239 |
| ENSG00000266714 | 1.21814531 |
| ENSG00000163864 | 1.216330171 |
| ENSG00000129757 | 1.214901453 |
| ENSG00000152127 | 1.212993133 |
| ENSG00000250834 | 1.211045643 |
| ENSG00000105974 | 1.210083186 |
| ENSG00000204380 | 1.208974705 |
| ENSG00000182986 | 1.208387711 |
| ENSG00000106031 | 1.207052632 |
| ENSG00000198440 | 1.203500687 |
| ENSG00000135905 | 1.202908556 |
| ENSG00000123405 | 1.201441923 |
| ENSG00000151715 | 1.19742271 |
| ENSG00000145012 | 1.19693446 |
| ENSG00000247157 | 1.194999552 |
| ENSG00000118046 | 1.193533804 |
| ENSG00000145730 | 1.191726569 |

| | |
|---|---|
| ENSG00000171345 | 1.189275003 |
| ENSG00000136826 | 1.188995061 |
| ENSG00000196466 | 1.186986501 |
| ENSG00000261115 | 1.185807103 |
| ENSG00000122644 | 1.181639336 |
| ENSG00000130270 | 1.178459761 |
| ENSG00000119125 | 1.17752637 |
| ENSG00000180855 | 1.177406362 |
| ENSG00000118513 | 1.173199541 |
| ENSG00000154240 | 1.172981314 |
| ENSG00000133135 | 1.171168491 |
| ENSG00000197497 | 1.16745109 |
| ENSG00000228828 | 1.164653424 |
| ENSG00000138670 | 1.162296846 |
| ENSG00000146411 | 1.160874064 |
| ENSG00000214870 | 1.159487627 |
| ENSG00000179981 | 1.154987614 |
| ENSG00000261324 | 1.154168242 |
| ENSG00000008256 | 1.151772326 |
| ENSG00000157111 | 1.151529998 |
| ENSG00000173598 | 1.150533579 |
| ENSG00000196781 | 1.149690022 |
| ENSG00000124813 | 1.147857753 |
| ENSG00000174307 | 1.147408931 |
| ENSG00000180914 | 1.14606565 |
| ENSG00000109771 | 1.145326336 |
| ENSG00000264644 | 1.144503431 |
| ENSG00000139629 | 1.141287037 |
| ENSG00000049130 | 1.13980908 |
| ENSG00000124882 | 1.139284935 |
| ENSG00000167767 | 1.138424302 |
| ENSG00000196542 | 1.136974913 |
| ENSG00000177721 | 1.131684659 |
| ENSG00000197608 | 1.130426374 |
| ENSG00000246228 | 1.129372069 |
| ENSG00000136531 | 1.128912738 |
| ENSG00000127334 | 1.126274787 |
| ENSG00000078804 | 1.12398816 |
| ENSG00000164684 | 1.123852424 |
| ENSG00000196154 | 1.123825479 |

| | |
|---|---|
| ENSG00000079337 | 1.122545346 |
| ENSG00000170689 | 1.122349861 |
| ENSG00000183671 | 1.121632841 |
| ENSG00000151726 | 1.119686317 |
| ENSG00000253485 | 1.113138069 |
| ENSG00000198482 | 1.111585779 |
| ENSG00000090776 | 1.111576481 |
| ENSG00000234771 | 1.111509315 |
| ENSG00000186642 | 1.105378074 |
| ENSG00000233589 | 1.10461814 |
| ENSG00000159166 | 1.101152595 |
| ENSG00000225361 | 1.099425958 |
| ENSG00000196975 | 1.097907867 |
| ENSG00000105963 | 1.097549694 |
| ENSG00000188483 | 1.096405172 |
| ENSG00000115414 | 1.096137135 |
| ENSG00000102043 | 1.096081797 |
| ENSG00000233559 | 1.091951854 |
| ENSG00000229152 | 1.090831677 |
| ENSG00000169247 | 1.090419409 |
| ENSG00000185101 | 1.089862173 |
| ENSG00000151806 | 1.08872718 |
| ENSG00000152894 | 1.087306091 |
| ENSG00000106258 | 1.087090648 |
| ENSG00000172264 | 1.085441759 |
| ENSG00000111057 | 1.085161346 |
| ENSG00000215807 | 1.084592281 |
| ENSG00000130997 | 1.084426386 |
| ENSG00000055118 | 1.084117153 |
| ENSG00000197951 | 1.083600201 |
| ENSG00000164867 | 1.082251524 |
| ENSG00000228158 | 1.079574284 |
| ENSG00000105464 | 1.079428049 |
| ENSG00000152229 | 1.078496387 |
| ENSG00000214207 | 1.078426898 |
| ENSG00000248905 | 1.077350357 |
| ENSG00000124766 | 1.075185166 |
| ENSG00000186976 | 1.073966326 |
| ENSG00000105792 | 1.068985472 |
| ENSG00000168453 | 1.067994307 |

| | |
|---|---|
| ENSG00000012171 | 1.067135387 |
| ENSG00000105519 | 1.066526422 |
| ENSG00000120885 | 1.065204283 |
| ENSG00000165092 | 1.064716889 |
| ENSG00000109586 | 1.060256966 |
| ENSG00000141510 | 1.057119355 |
| ENSG00000272077 | 1.056984468 |
| ENSG00000091879 | 1.053294712 |
| ENSG00000163545 | 1.051230362 |
| ENSG00000184675 | 1.050997037 |
| ENSG00000186193 | 1.050283263 |
| ENSG00000100100 | 1.049376239 |
| ENSG00000173334 | 1.048809311 |
| ENSG00000099812 | 1.048323778 |
| ENSG00000215089 | 1.046142049 |
| ENSG00000256043 | 1.04609901 |
| ENSG00000095932 | 1.045683724 |
| ENSG00000156510 | 1.045068863 |
| ENSG00000092964 | 1.044295376 |
| ENSG00000235275 | 1.044226525 |
| ENSG00000180438 | 1.042439028 |
| ENSG00000213949 | 1.041767557 |
| ENSG00000163818 | 1.040566168 |
| ENSG00000129451 | 1.04040322 |
| ENSG00000197044 | 1.039276277 |
| ENSG00000180801 | 1.038195107 |
| ENSG00000221890 | 1.037037283 |
| ENSG00000103064 | 1.036775331 |
| ENSG00000179455 | 1.035243872 |
| ENSG00000224818 | 1.033485481 |
| ENSG00000136842 | 1.032734782 |
| ENSG00000168785 | 1.024636427 |
| ENSG00000227124 | 1.022910817 |
| ENSG00000135821 | 1.021181694 |
| ENSG00000198774 | 1.021171153 |
| ENSG00000111110 | 1.01973223 |
| ENSG00000182742 | 1.016320158 |
| ENSG00000110841 | 1.008403695 |
| ENSG00000158352 | 1.000947237 |
| ENSG00000217889 | 1.000738666 |

| | |
|---|---|
| ENSG00000101460 | 0.999341588 |
| ENSG00000213943 | 0.998989944 |
| ENSG00000135046 | 0.997782906 |
| ENSG00000182319 | 0.996497088 |
| ENSG00000107719 | 0.994771936 |
| ENSG00000203780 | 0.994671022 |
| ENSG00000088367 | 0.992091788 |
| ENSG00000213493 | 0.991870688 |
| ENSG00000140526 | 0.991540729 |
| ENSG00000253159 | 0.987166038 |
| ENSG00000163132 | 0.98665485 |
| ENSG00000049449 | 0.985913952 |
| ENSG00000101846 | 0.984823948 |
| ENSG00000231119 | 0.984491123 |
| ENSG00000251675 | 0.983270624 |
| ENSG00000117984 | 0.982288945 |
| ENSG00000081665 | 0.981906967 |
| ENSG00000259820 | 0.981409183 |
| ENSG00000007372 | 0.981105712 |
| ENSG00000163803 | 0.980834779 |
| ENSG00000177144 | 0.979176628 |
| ENSG00000225345 | 0.978010321 |
| ENSG00000186451 | 0.977042039 |
| ENSG00000184635 | 0.973402397 |
| ENSG00000165895 | 0.972976329 |
| ENSG00000164764 | 0.972043545 |
| ENSG00000120675 | 0.971760903 |
| ENSG00000121769 | 0.971440434 |
| ENSG00000175611 | 0.970918602 |
| ENSG00000172296 | 0.970031927 |
| ENSG00000267475 | 0.968757852 |
| ENSG00000259976 | 0.96723455 |
| ENSG00000184956 | 0.964287318 |
| ENSG00000168306 | 0.963543621 |
| ENSG00000214455 | 0.959626017 |
| ENSG00000161551 | 0.959192876 |
| ENSG00000141293 | 0.958480752 |
| ENSG00000101665 | 0.958462924 |
| ENSG00000232928 | 0.957128372 |
| ENSG00000213958 | 0.955364552 |

| | |
|---|---|
| ENSG00000223650 | 0.952755558 |
| ENSG00000099860 | 0.95241737 |
| ENSG00000142149 | 0.951280108 |
| ENSG00000136883 | 0.949381677 |
| ENSG00000267481 | 0.948992352 |
| ENSG00000153714 | 0.948443933 |
| ENSG00000170647 | 0.945889396 |
| ENSG00000207650 | 0.943501462 |
| ENSG00000184995 | 0.943499424 |
| ENSG00000091409 | 0.943155889 |
| ENSG00000039139 | 0.943006057 |
| ENSG00000019549 | 0.942096547 |
| ENSG00000100311 | 0.941461622 |
| ENSG00000137642 | 0.941266112 |
| ENSG00000181031 | 0.939606786 |
| ENSG00000143466 | 0.939283351 |
| ENSG00000160223 | 0.939009608 |
| ENSG00000179978 | 0.937128624 |
| ENSG00000163513 | 0.936685838 |
| ENSG00000197020 | 0.935935469 |
| ENSG00000125398 | 0.935136179 |
| ENSG00000198590 | 0.933887954 |
| ENSG00000117394 | 0.933073141 |
| ENSG00000136059 | 0.932677415 |
| ENSG00000101144 | 0.932532304 |
| ENSG00000141738 | 0.931979852 |
| ENSG00000198466 | 0.931700681 |
| ENSG00000229953 | 0.928375819 |
| ENSG00000124201 | 0.927854347 |
| ENSG00000173210 | 0.925291213 |
| ENSG00000165105 | 0.92454584 |
| ENSG00000165646 | 0.924356412 |
| ENSG00000136169 | 0.92355818 |
| ENSG00000112699 | 0.922677994 |
| ENSG00000130402 | 0.921557655 |
| ENSG00000196436 | 0.918933262 |
| ENSG00000171617 | 0.918634108 |
| ENSG00000070882 | 0.917193813 |
| ENSG00000163131 | 0.915588938 |
| ENSG00000234737 | 0.914367347 |

| | |
|---|---|
| ENSG00000148926 | 0.913936683 |
| ENSG00000186567 | 0.912487716 |
| ENSG00000095637 | 0.911661437 |
| ENSG00000111252 | 0.911535353 |
| ENSG00000234444 | 0.910627914 |
| ENSG00000227036 | 0.908786268 |
| ENSG00000256087 | 0.904062166 |
| ENSG00000129422 | 0.903154619 |
| ENSG00000214012 | 0.901733163 |
| ENSG00000127863 | 0.900998527 |
| ENSG00000148400 | 0.899951716 |
| ENSG00000133808 | 0.899919454 |
| ENSG00000139354 | 0.898168612 |
| ENSG00000151376 | 0.897585898 |
| ENSG00000164300 | 0.897322751 |
| ENSG00000101311 | 0.896936729 |
| ENSG00000110944 | 0.896451989 |
| ENSG00000197296 | 0.893851899 |
| ENSG00000169583 | 0.893709765 |
| ENSG00000102908 | 0.893388101 |
| ENSG00000134909 | 0.893310932 |
| ENSG00000080503 | 0.892315403 |
| ENSG00000256771 | 0.891333845 |
| ENSG00000006047 | 0.891111994 |
| ENSG00000162543 | 0.890556335 |
| ENSG00000121957 | 0.889824159 |
| ENSG00000175662 | 0.888651304 |
| ENSG00000149948 | 0.887486557 |
| ENSG00000106829 | 0.884848069 |
| ENSG00000166349 | 0.88477331 |
| ENSG00000135205 | 0.884327526 |
| ENSG00000272373 | 0.883674985 |
| ENSG00000167778 | 0.8810357 |
| ENSG00000160789 | 0.880976591 |
| ENSG00000163527 | 0.880175986 |
| ENSG00000187210 | 0.879590676 |
| ENSG00000079819 | 0.878867562 |
| ENSG00000213626 | 0.878266913 |
| ENSG00000095321 | 0.878140238 |
| ENSG00000101353 | 0.876856568 |

| | |
|---|---|
| ENSG00000184384 | 0.876431768 |
| ENSG00000056558 | 0.876373852 |
| ENSG00000181722 | 0.875699148 |
| ENSG00000181381 | 0.873818176 |
| ENSG00000204956 | 0.872496657 |
| ENSG00000213462 | 0.871322237 |
| ENSG00000225572 | 0.871031675 |
| ENSG00000176842 | 0.87019842 |
| ENSG00000170919 | 0.870004254 |
| ENSG00000120324 | 0.869685347 |
| ENSG00000164951 | 0.868264727 |
| ENSG00000139832 | 0.867810106 |
| ENSG00000214900 | 0.866524143 |
| ENSG00000186594 | 0.864413777 |
| ENSG00000135363 | 0.86418306 |
| ENSG00000187699 | 0.864181406 |
| ENSG00000185551 | 0.863694772 |
| ENSG00000271927 | 0.860778362 |
| ENSG00000135049 | 0.860177563 |
| ENSG00000152582 | 0.860073394 |
| ENSG00000151572 | 0.860040876 |
| ENSG00000187105 | 0.859911976 |
| ENSG00000105971 | 0.859569642 |
| ENSG00000120708 | 0.85793456 |
| ENSG00000260032 | 0.857881216 |
| ENSG00000270959 | 0.857771449 |
| ENSG00000163820 | 0.857758048 |
| ENSG00000196912 | 0.85646799 |
| ENSG00000115648 | 0.856260646 |
| ENSG00000186792 | 0.855417918 |
| ENSG00000170385 | 0.853748414 |
| ENSG00000176920 | 0.852131838 |
| ENSG00000144283 | 0.850708712 |
| ENSG00000159216 | 0.850570246 |
| ENSG00000255874 | 0.849337977 |
| ENSG00000174951 | 0.84723451 |
| ENSG00000185567 | 0.847211153 |
| ENSG00000009950 | 0.846570603 |
| ENSG00000168961 | 0.843511699 |
| ENSG00000158470 | 0.840705388 |

| | |
|---|---|
| ENSG00000196335 | 0.840542972 |
| ENSG00000155111 | 0.840344021 |
| ENSG00000231697 | 0.838387135 |
| ENSG00000002834 | 0.836410971 |
| ENSG00000134954 | 0.835260205 |
| ENSG00000016391 | 0.833864791 |
| ENSG00000159173 | 0.833588265 |
| ENSG00000253368 | 0.832590791 |
| ENSG00000006459 | 0.832286756 |
| ENSG00000174370 | 0.831424566 |
| ENSG00000050344 | 0.8305379 |
| ENSG00000249437 | 0.829871009 |
| ENSG00000119862 | 0.828804915 |
| ENSG00000064932 | 0.828086843 |
| ENSG00000182141 | 0.826941811 |
| ENSG00000159905 | 0.826779183 |
| ENSG00000158023 | 0.826458462 |
| ENSG00000081923 | 0.825742108 |
| ENSG00000069812 | 0.825517208 |
| ENSG00000129946 | 0.823240636 |
| ENSG00000178826 | 0.821732578 |
| ENSG00000122547 | 0.821637581 |
| ENSG00000138771 | 0.821479389 |
| ENSG00000173262 | 0.820467016 |
| ENSG00000135048 | 0.82043178 |
| ENSG00000242602 | 0.819619886 |
| ENSG00000087074 | 0.819408738 |
| ENSG00000184678 | 0.818701667 |
| ENSG00000106460 | 0.817999072 |
| ENSG00000170145 | 0.817718696 |
| ENSG00000196535 | 0.817673451 |
| ENSG00000124942 | 0.817360516 |
| ENSG00000171552 | 0.815193095 |
| ENSG00000199024 | 0.815066021 |
| ENSG00000224429 | 0.814202227 |
| ENSG00000163995 | 0.813499008 |
| ENSG00000115042 | 0.81278951 |
| ENSG00000151746 | 0.812381965 |
| ENSG00000139438 | 0.811549675 |
| ENSG00000106078 | 0.811423729 |

| | |
|---|---|
| ENSG00000238105 | 0.811237352 |
| ENSG00000261534 | 0.809694462 |
| ENSG00000124788 | 0.808693611 |
| ENSG00000100344 | 0.808000592 |
| ENSG00000130956 | 0.80746167 |
| ENSG00000106799 | 0.807011726 |
| ENSG00000186352 | 0.806851433 |
| ENSG00000151743 | 0.806363169 |
| ENSG00000130477 | 0.806251403 |
| ENSG00000131242 | 0.804708226 |
| ENSG00000224093 | 0.804181942 |
| ENSG00000227638 | 0.803424112 |
| ENSG00000161328 | 0.803321558 |
| ENSG00000165650 | 0.80279381 |
| ENSG00000251634 | 0.802474893 |
| ENSG00000064651 | 0.80235983 |
| ENSG00000206712 | 0.801862541 |
| ENSG00000206530 | 0.801225475 |
| ENSG00000203813 | 0.800631909 |
| ENSG00000271643 | 0.797179462 |
| ENSG00000196935 | 0.796663614 |
| ENSG00000151117 | 0.795964861 |
| ENSG00000134030 | 0.795482531 |
| ENSG00000170542 | 0.795073927 |
| ENSG00000145901 | 0.794836686 |
| ENSG00000229127 | 0.794802948 |
| ENSG00000135503 | 0.791018525 |
| ENSG00000214439 | 0.789204514 |
| ENSG00000251432 | 0.788900915 |
| ENSG00000272405 | 0.78882711 |
| ENSG00000205482 | 0.785813779 |
| ENSG00000205238 | 0.78483797 |
| ENSG00000119408 | 0.78413258 |
| ENSG00000254876 | 0.783982562 |
| ENSG00000103381 | 0.782749122 |
| ENSG00000112033 | 0.781831121 |
| ENSG00000065609 | 0.780711984 |
| ENSG00000163697 | 0.780165181 |
| ENSG00000213977 | 0.780154815 |
| ENSG00000178038 | 0.779680431 |

| | |
|---|---|
| ENSG00000154930 | 0.779049812 |
| ENSG00000092068 | 0.779015356 |
| ENSG00000174099 | 0.777892059 |
| ENSG00000105849 | 0.777398351 |
| ENSG00000154035 | 0.777276685 |
| ENSG00000253636 | 0.777244048 |
| ENSG00000096696 | 0.777040572 |
| ENSG00000111907 | 0.77625683 |
| ENSG00000183579 | 0.774815539 |
| ENSG00000186704 | 0.774728386 |
| ENSG00000186300 | 0.773292519 |
| ENSG00000188868 | 0.771583118 |
| ENSG00000233016 | 0.77147934 |
| ENSG00000165868 | 0.770945447 |
| ENSG00000170017 | 0.769761886 |
| ENSG00000172057 | 0.767497566 |
| ENSG00000132170 | 0.767232973 |
| ENSG00000261167 | 0.767213142 |
| ENSG00000137962 | 0.767111565 |
| ENSG00000235109 | 0.765577542 |
| ENSG00000135407 | 0.765241339 |
| ENSG00000137124 | 0.765091192 |
| ENSG00000114796 | 0.764012556 |
| ENSG00000179532 | 0.76305217 |
| ENSG00000172006 | 0.762985465 |
| ENSG00000273148 | 0.761910885 |
| ENSG00000204334 | 0.761175202 |
| ENSG00000250903 | 0.760794978 |
| ENSG00000245213 | 0.75979996 |
| ENSG00000167755 | 0.759793102 |
| ENSG00000128872 | 0.759597905 |
| ENSG00000144677 | 0.759562806 |
| ENSG00000160401 | 0.757982673 |
| ENSG00000171729 | 0.757974034 |
| ENSG00000106012 | 0.757906996 |
| ENSG00000246273 | 0.756971528 |
| ENSG00000221995 | 0.756292769 |
| ENSG00000133106 | 0.756152298 |
| ENSG00000174738 | 0.756026073 |
| ENSG00000196814 | 0.755626772 |

| | |
|---|---|
| ENSG00000108771 | 0.753808571 |
| ENSG00000261005 | 0.752773826 |
| ENSG00000272335 | 0.752315264 |
| ENSG00000146776 | 0.752024387 |
| ENSG00000186047 | 0.74817121 |
| ENSG00000197535 | 0.74804743 |
| ENSG00000197872 | 0.747199153 |
| ENSG00000166016 | 0.746797318 |
| ENSG00000174808 | 0.746365031 |
| ENSG00000197142 | 0.746234436 |
| ENSG00000103018 | 0.743604974 |
| ENSG00000180257 | 0.743285703 |
| ENSG00000136114 | 0.743177986 |
| ENSG00000267283 | 0.742676812 |
| ENSG00000196074 | 0.741900276 |
| ENSG00000160188 | 0.741410316 |
| ENSG00000167680 | 0.740711279 |
| ENSG00000166888 | 0.740709871 |
| ENSG00000167757 | 0.739900882 |
| ENSG00000165115 | 0.739136315 |
| ENSG00000196456 | 0.738760058 |
| ENSG00000157557 | 0.738474546 |
| ENSG00000179104 | 0.737512261 |
| ENSG00000245532 | 0.736967682 |
| ENSG00000245849 | 0.736383232 |
| ENSG00000206503 | 0.735859178 |
| ENSG00000164050 | 0.735326647 |
| ENSG00000158062 | 0.734582482 |
| ENSG00000197409 | 0.734395022 |
| ENSG00000179909 | 0.733328393 |
| ENSG00000103351 | 0.732973172 |
| ENSG00000106785 | 0.732861811 |
| ENSG00000088448 | 0.732761398 |
| ENSG00000010818 | 0.73233065 |
| ENSG00000175104 | 0.731653855 |
| ENSG00000132692 | 0.731158733 |
| ENSG00000092758 | 0.729846455 |
| ENSG00000159263 | 0.729079604 |
| ENSG00000225205 | 0.728536119 |
| ENSG00000137462 | 0.726565968 |

| | |
|---|---|
| ENSG00000259456 | 0.726434848 |
| ENSG00000104783 | 0.725693387 |
| ENSG00000197837 | 0.725475588 |
| ENSG00000234684 | 0.725102918 |
| ENSG00000182963 | 0.724224604 |
| ENSG00000160209 | 0.724069754 |
| ENSG00000255103 | 0.723882785 |
| ENSG00000204604 | 0.723877937 |
| ENSG00000170340 | 0.723333337 |
| ENSG00000228463 | 0.721831731 |
| ENSG00000136052 | 0.721824484 |
| ENSG00000123870 | 0.721136933 |
| ENSG00000260630 | 0.721002846 |
| ENSG00000124098 | 0.719921394 |
| ENSG00000121716 | 0.71963664 |
| ENSG00000173905 | 0.7193351 |
| ENSG00000125637 | 0.719144205 |
| ENSG00000267041 | 0.7190294 |
| ENSG00000175592 | 0.718771647 |
| ENSG00000188659 | 0.717335164 |
| ENSG00000123104 | 0.715711457 |
| ENSG00000147862 | 0.715283458 |
| ENSG00000151414 | 0.715188343 |
| ENSG00000100290 | 0.715116314 |
| ENSG00000085741 | 0.715027229 |
| ENSG00000272168 | 0.714123806 |
| ENSG00000168273 | 0.713709713 |
| ENSG00000251474 | 0.713382246 |
| ENSG00000259001 | 0.71333539 |
| ENSG00000186638 | 0.71264362 |
| ENSG00000073605 | 0.712375343 |
| ENSG00000123836 | 0.712305015 |
| ENSG00000250015 | 0.712282661 |
| ENSG00000103249 | 0.712225333 |
| ENSG00000147853 | 0.712041811 |
| ENSG00000110921 | 0.711894685 |
| ENSG00000109452 | 0.71181184 |
| ENSG00000197536 | 0.710895156 |
| ENSG00000189120 | 0.710086561 |
| ENSG00000165194 | 0.70848761 |

| | |
|---|---|
| ENSG00000135373 | 0.707693163 |
| ENSG00000132793 | 0.707563452 |
| ENSG00000267680 | 0.706616329 |
| ENSG00000255455 | 0.705727713 |
| ENSG00000235162 | 0.705439168 |
| ENSG00000257122 | 0.705003394 |
| ENSG00000264112 | 0.70492747 |
| ENSG00000203880 | 0.704915231 |
| ENSG00000125971 | 0.704326137 |
| ENSG00000143630 | 0.703883619 |
| ENSG00000078401 | 0.703807553 |
| ENSG00000149596 | 0.70303179 |
| ENSG00000259953 | 0.702839998 |
| ENSG00000197070 | 0.701425179 |
| ENSG00000164845 | 0.700676033 |
| ENSG00000154556 | 0.699334199 |
| ENSG00000101421 | 0.698257554 |
| ENSG00000184545 | 0.697042859 |
| ENSG00000116260 | 0.696424344 |
| ENSG00000171843 | 0.696414546 |
| ENSG00000197329 | 0.695301699 |
| ENSG00000231731 | 0.695231854 |
| ENSG00000171045 | 0.694981069 |
| ENSG00000198729 | 0.694886982 |
| ENSG00000059804 | 0.694126527 |
| ENSG00000182923 | 0.693242376 |
| ENSG00000273079 | 0.692526606 |
| ENSG00000006704 | 0.691835551 |
| ENSG00000242110 | 0.691427184 |
| ENSG00000133816 | 0.691030526 |
| ENSG00000128342 | 0.690356497 |
| ENSG00000125844 | 0.689402605 |
| ENSG00000117115 | 0.68940049 |
| ENSG00000253598 | 0.689261746 |
| ENSG00000118620 | 0.688561764 |
| ENSG00000272093 | 0.687579628 |
| ENSG00000271204 | 0.687418646 |
| ENSG00000183856 | 0.687397114 |
| ENSG00000140948 | 0.687380495 |
| ENSG00000172765 | 0.686993604 |

| | |
|---|---|
| ENSG00000132669 | 0.686666693 |
| ENSG00000135924 | 0.686181917 |
| ENSG00000109501 | 0.686163689 |
| ENSG00000106366 | 0.685387055 |
| ENSG00000259397 | 0.684153235 |
| ENSG00000160796 | 0.683430341 |
| ENSG00000101439 | 0.683341751 |
| ENSG00000008441 | 0.682823258 |
| ENSG00000235454 | 0.682521974 |
| ENSG00000167508 | 0.68242244 |
| ENSG00000223749 | 0.681885194 |
| ENSG00000135976 | 0.680666659 |
| ENSG00000092969 | 0.680145872 |
| ENSG00000168528 | 0.680024796 |
| ENSG00000170873 | 0.679720318 |
| ENSG00000144369 | 0.67749078 |
| ENSG00000225485 | 0.675906434 |
| ENSG00000153976 | 0.675297149 |
| ENSG00000169964 | 0.674414537 |
| ENSG00000106546 | 0.674172548 |
| ENSG00000163617 | 0.673994449 |
| ENSG00000181350 | 0.673831185 |
| ENSG00000146574 | 0.673557825 |
| ENSG00000102189 | 0.673425538 |
| ENSG00000110092 | 0.673038873 |
| ENSG00000142192 | 0.672911766 |
| ENSG00000182667 | 0.672676845 |
| ENSG00000106006 | 0.672057696 |
| ENSG00000062370 | 0.671828872 |
| ENSG00000160216 | 0.671255234 |
| ENSG00000099804 | 0.670910874 |
| ENSG00000235865 | 0.668715374 |
| ENSG00000242767 | 0.668562403 |
| ENSG00000135596 | 0.667966032 |
| ENSG00000273033 | 0.667363353 |
| ENSG00000164087 | 0.666958125 |
| ENSG00000140299 | 0.666808457 |
| ENSG00000106852 | 0.66666119 |
| ENSG00000179431 | 0.666444234 |
| ENSG00000116679 | 0.665794161 |

| | |
|---|---|
| ENSG00000118689 | 0.664284902 |
| ENSG00000111696 | 0.663080362 |
| ENSG00000164056 | 0.662854441 |
| ENSG00000183741 | 0.662777807 |
| ENSG00000114631 | 0.66267642 |
| ENSG00000077984 | 0.662372163 |
| ENSG00000169692 | 0.662200312 |
| ENSG00000145632 | 0.661921407 |
| ENSG00000180672 | 0.66145101 |
| ENSG00000007516 | 0.660701781 |
| ENSG00000196693 | 0.659921208 |
| ENSG00000267002 | 0.659569265 |
| ENSG00000236876 | 0.659357029 |
| ENSG00000125266 | 0.658819625 |
| ENSG00000109787 | 0.658539543 |
| ENSG00000241170 | 0.658461899 |
| ENSG00000233006 | 0.658456303 |
| ENSG00000136802 | 0.657923578 |
| ENSG00000259243 | 0.65779105 |
| ENSG00000157985 | 0.65757711 |
| ENSG00000148339 | 0.657328752 |
| ENSG00000125731 | 0.656898964 |
| ENSG00000214239 | 0.656254558 |
| ENSG00000148090 | 0.65609461 |
| ENSG00000165661 | 0.655661589 |
| ENSG00000271976 | 0.655483405 |
| ENSG00000095383 | 0.65529389 |
| ENSG00000245680 | 0.65488143 |
| ENSG00000183718 | 0.654755101 |
| ENSG00000267838 | 0.654636375 |
| ENSG00000214353 | 0.654590895 |
| ENSG00000145087 | 0.654404106 |
| ENSG00000189319 | 0.654172812 |
| ENSG00000204920 | 0.653868385 |
| ENSG00000247498 | 0.653713909 |
| ENSG00000101333 | 0.653497584 |
| ENSG00000115963 | 0.653484376 |
| ENSG00000139163 | 0.652960616 |
| ENSG00000070404 | 0.652832039 |
| ENSG00000152104 | 0.652772998 |

| | |
|---|---|
| ENSG00000050405 | 0.65221534 |
| ENSG00000100065 | 0.651807989 |
| ENSG00000246334 | 0.651209213 |
| ENSG00000232859 | 0.650978493 |
| ENSG00000198000 | 0.650491408 |
| ENSG00000112144 | 0.649678433 |
| ENSG00000197933 | 0.649119881 |
| ENSG00000261526 | 0.64868437 |
| ENSG00000142910 | 0.648202892 |
| ENSG00000105982 | 0.648146759 |
| ENSG00000167600 | 0.647965926 |
| ENSG00000106261 | 0.647667547 |
| ENSG00000124839 | 0.647294641 |
| ENSG00000186166 | 0.64666855 |
| ENSG00000203865 | 0.645774125 |
| ENSG00000123146 | 0.645757143 |
| ENSG00000114735 | 0.645621801 |
| ENSG00000204267 | 0.645042858 |
| ENSG00000149292 | 0.644563766 |
| ENSG00000139044 | 0.643450516 |
| ENSG00000137073 | 0.643197171 |
| ENSG00000232811 | 0.642916207 |
| ENSG00000167106 | 0.642789261 |
| ENSG00000145675 | 0.642753135 |
| ENSG00000267106 | 0.642553962 |
| ENSG00000240891 | 0.642543557 |
| ENSG00000148341 | 0.642295331 |
| ENSG00000236824 | 0.64226549 |
| ENSG00000160271 | 0.64209261 |
| ENSG00000005238 | 0.641878666 |
| ENSG00000188107 | 0.641533845 |
| ENSG00000099875 | 0.641494012 |
| ENSG00000145349 | 0.641288891 |
| ENSG00000243926 | 0.640220893 |
| ENSG00000165905 | 0.6399414 |
| ENSG00000184227 | 0.63969287 |
| ENSG00000226381 | 0.639420226 |
| ENSG00000021300 | 0.639389552 |
| ENSG00000144674 | 0.638959465 |
| ENSG00000250069 | 0.638864198 |

| | |
|---|---|
| ENSG00000101442 | 0.638517249 |
| ENSG00000131374 | 0.638335916 |
| ENSG00000175772 | 0.637948951 |
| ENSG00000171084 | 0.637806348 |
| ENSG00000166667 | 0.636964487 |
| ENSG00000114853 | 0.636835835 |
| ENSG00000198642 | 0.636210618 |
| ENSG00000041982 | 0.63611243 |
| ENSG00000185040 | 0.634480327 |
| ENSG00000141933 | 0.634356909 |
| ENSG00000148411 | 0.634152475 |
| ENSG00000144645 | 0.633982672 |
| ENSG00000167779 | 0.632018879 |
| ENSG00000259673 | 0.631486152 |
| ENSG00000205542 | 0.630879389 |
| ENSG00000171606 | 0.629890426 |
| ENSG00000108292 | 0.629341412 |
| ENSG00000133789 | 0.629324432 |
| ENSG00000178386 | 0.629251917 |
| ENSG00000007384 | 0.6288829 |
| ENSG00000205808 | 0.628602125 |
| ENSG00000049883 | 0.627838046 |
| ENSG00000127955 | 0.627083275 |
| ENSG00000143344 | 0.626488623 |
| ENSG00000120800 | 0.626226675 |
| ENSG00000155918 | 0.626089634 |
| ENSG00000203709 | 0.625742528 |
| ENSG00000104892 | 0.624956164 |
| ENSG00000114779 | 0.624824183 |
| ENSG00000272033 | 0.624412812 |
| ENSG00000128833 | 0.623889949 |
| ENSG00000189143 | 0.623839864 |
| ENSG00000114738 | 0.623517341 |
| ENSG00000164323 | 0.623427998 |
| ENSG00000271971 | 0.623165482 |
| ENSG00000104936 | 0.622907265 |
| ENSG00000172936 | 0.622471454 |
| ENSG00000140455 | 0.621786707 |
| ENSG00000214176 | 0.621415191 |
| ENSG00000145113 | 0.621106786 |

| | |
|---|---|
| ENSG00000256383 | 0.62085357 |
| ENSG00000212232 | 0.620473643 |
| ENSG00000205220 | 0.620449489 |
| ENSG00000011523 | 0.620022465 |
| ENSG00000124177 | 0.6199317 |
| ENSG00000049759 | 0.619770406 |
| ENSG00000128000 | 0.619580103 |
| ENSG00000143147 | 0.619398174 |
| ENSG00000103534 | 0.617346963 |
| ENSG00000197457 | 0.616626109 |
| ENSG00000101384 | 0.616411032 |
| ENSG00000239388 | 0.616365024 |
| ENSG00000146021 | 0.616283293 |
| ENSG00000196597 | 0.61616875 |
| ENSG00000246705 | 0.615234322 |
| ENSG00000164920 | 0.614955103 |
| ENSG00000215769 | 0.614898964 |
| ENSG00000133639 | 0.614872135 |
| ENSG00000223891 | 0.61471937 |
| ENSG00000262944 | 0.614615553 |
| ENSG00000136877 | 0.614311264 |
| ENSG00000186448 | 0.614265162 |
| ENSG00000002822 | 0.612691462 |
| ENSG00000144559 | 0.612520159 |
| ENSG00000273061 | 0.610513703 |
| ENSG00000173678 | 0.610297444 |
| ENSG00000260942 | 0.610224539 |
| ENSG00000085514 | 0.609939896 |
| ENSG00000197580 | 0.609912805 |
| ENSG00000234819 | 0.609900363 |
| ENSG00000198538 | 0.609731221 |
| ENSG00000233993 | 0.608426233 |
| ENSG00000007376 | 0.607912316 |
| ENSG00000114745 | 0.607806103 |
| ENSG00000153560 | 0.607732226 |
| ENSG00000183087 | 0.607370941 |
| ENSG00000172361 | 0.607262627 |
| ENSG00000143850 | 0.606835724 |
| ENSG00000152763 | 0.606225751 |
| ENSG00000237118 | 0.605904216 |

| | |
|---|---|
| ENSG00000185774 | 0.605478549 |
| ENSG00000267710 | 0.605401343 |
| ENSG00000267519 | 0.605106138 |
| ENSG00000152223 | 0.60487817 |
| ENSG00000167693 | 0.603985713 |
| ENSG00000254595 | 0.603497501 |
| ENSG00000160285 | 0.603094678 |
| ENSG00000129474 | 0.602549088 |
| ENSG00000131370 | 0.601744284 |
| ENSG00000125772 | 0.601648753 |
| ENSG00000197724 | 0.601636 |
| ENSG00000141994 | 0.601605444 |
| ENSG00000206337 | 0.600792543 |
| ENSG00000130054 | 0.600746879 |
| ENSG00000215066 | 0.60067813 |
| ENSG00000132359 | 0.600372386 |
| ENSG00000213859 | 0.600033089 |
| ENSG00000198780 | 0.600020977 |
| ENSG00000198208 | 0.600016896 |
| ENSG00000214078 | 0.599423437 |
| ENSG00000143429 | 0.598675016 |
| ENSG00000253854 | 0.598388195 |
| ENSG00000230163 | 0.597871306 |
| ENSG00000247400 | 0.597832616 |
| ENSG00000165121 | 0.597442486 |
| ENSG00000147174 | 0.596089772 |
| ENSG00000204673 | 0.595591686 |
| ENSG00000171241 | 0.595011028 |
| ENSG00000222041 | 0.594767816 |
| ENSG00000124228 | 0.594699599 |
| ENSG00000228797 | 0.594187754 |
| ENSG00000228393 | 0.594052218 |
| ENSG00000171222 | 0.594000661 |
| ENSG00000273356 | 0.593322013 |
| ENSG00000167306 | 0.593104568 |
| ENSG00000117036 | 0.592725018 |
| ENSG00000180891 | 0.592086333 |
| ENSG00000139734 | 0.591947406 |
| ENSG00000104419 | 0.591339108 |
| ENSG00000108270 | 0.591283131 |

| | |
|---|---|
| ENSG00000165171 | 0.590043222 |
| ENSG00000109911 | 0.589371719 |
| ENSG00000137266 | 0.588937973 |
| ENSG00000110851 | 0.588684615 |
| ENSG00000136936 | 0.588520374 |
| ENSG00000136830 | 0.588454652 |
| ENSG00000198785 | 0.588330535 |
| ENSG00000167562 | 0.587917152 |
| ENSG00000213073 | 0.587893645 |
| ENSG00000187951 | 0.587620456 |
| ENSG00000123485 | 0.587418072 |
| ENSG00000198858 | 0.587122804 |
| ENSG00000270018 | 0.586886072 |
| ENSG00000141682 | 0.586292246 |
| ENSG00000101266 | 0.585899752 |
| ENSG00000129282 | 0.585801069 |
| ENSG00000168077 | 0.585713334 |
| ENSG00000133624 | 0.584900005 |
| ENSG00000173041 | 0.584861842 |
| ENSG00000152558 | 0.584774598 |
| ENSG00000166762 | 0.584511796 |
| ENSG00000182472 | 0.584387572 |
| ENSG00000165684 | 0.584092755 |
| ENSG00000076826 | 0.583991893 |
| ENSG00000127948 | 0.583906007 |
| ENSG00000239887 | 0.583795204 |
| ENSG00000205281 | 0.583766901 |
| ENSG00000262758 | 0.583688676 |
| ENSG00000173801 | 0.58361055 |
| ENSG00000081377 | 0.583002989 |
| ENSG00000188315 | 0.582939467 |
| ENSG00000196247 | 0.582014711 |
| ENSG00000134815 | 0.581775658 |
| ENSG00000205084 | 0.581713885 |
| ENSG00000170684 | 0.581627835 |
| ENSG00000154639 | 0.581485112 |
| ENSG00000197766 | 0.581295264 |
| ENSG00000258947 | 0.581191699 |
| ENSG00000271020 | 0.58073254 |
| ENSG00000160218 | 0.580017111 |

| | |
|---|---|
| ENSG00000157064 | 0.579980832 |
| ENSG00000164506 | 0.578959747 |
| ENSG00000048740 | 0.578663565 |
| ENSG00000174917 | 0.578103237 |
| ENSG00000197385 | 0.57800056 |
| ENSG00000136206 | 0.577683289 |
| ENSG00000125257 | 0.577651431 |
| ENSG00000231566 | 0.57667751 |
| ENSG00000149177 | 0.576532199 |
| ENSG00000136925 | 0.576089707 |
| ENSG00000180332 | 0.575588619 |
| ENSG00000248483 | 0.575446089 |
| ENSG00000157578 | 0.575192314 |
| ENSG00000106823 | 0.574786472 |
| ENSG00000168078 | 0.574743922 |
| ENSG00000179912 | 0.57470427 |
| ENSG00000259248 | 0.574156103 |
| ENSG00000161714 | 0.574019925 |
| ENSG00000010810 | 0.572750071 |
| ENSG00000213020 | 0.5724611 |
| ENSG00000189190 | 0.571739941 |
| ENSG00000137880 | 0.571691649 |
| ENSG00000125875 | 0.571624203 |
| ENSG00000259583 | 0.571576759 |
| ENSG00000148356 | 0.571152264 |
| ENSG00000161326 | 0.570519599 |
| ENSG00000107731 | 0.570335112 |
| ENSG00000125814 | 0.570030031 |
| ENSG00000178078 | 0.56950001 |
| ENSG00000233971 | 0.569343453 |
| ENSG00000119139 | 0.569230074 |
| ENSG00000198853 | 0.569197666 |
| ENSG00000254598 | 0.56908493 |
| ENSG00000236570 | 0.56835095 |
| ENSG00000272356 | 0.567946094 |
| ENSG00000101019 | 0.567907033 |
| ENSG00000047932 | 0.56782984 |
| ENSG00000271452 | 0.567482762 |
| ENSG00000196275 | 0.567370934 |
| ENSG00000164828 | 0.567072424 |

| | |
|---|---|
| ENSG00000083223 | 0.566446805 |
| ENSG00000214022 | 0.566366208 |
| ENSG00000168062 | 0.566283255 |
| ENSG00000121350 | 0.565780808 |
| ENSG00000138772 | 0.565597121 |
| ENSG00000144791 | 0.565482118 |
| ENSG00000261512 | 0.564333563 |
| ENSG00000136141 | 0.564044823 |
| ENSG00000118922 | 0.563469917 |
| ENSG00000156970 | 0.56343314 |
| ENSG00000146410 | 0.563389767 |
| ENSG00000167114 | 0.563381495 |
| ENSG00000189283 | 0.56321978 |
| ENSG00000132274 | 0.563134752 |
| ENSG00000196914 | 0.562829351 |
| ENSG00000051620 | 0.561934957 |
| ENSG00000235768 | 0.561072894 |
| ENSG00000225855 | 0.56061143 |
| ENSG00000100983 | 0.560500585 |
| ENSG00000152443 | 0.560315614 |
| ENSG00000172888 | 0.559766662 |
| ENSG00000177106 | 0.559647074 |
| ENSG00000170791 | 0.55956601 |
| ENSG00000180902 | 0.559302728 |
| ENSG00000165698 | 0.559123501 |
| ENSG00000261455 | 0.558810729 |
| ENSG00000136098 | 0.558298904 |
| ENSG00000111145 | 0.558285255 |
| ENSG00000135002 | 0.558144142 |
| ENSG00000185480 | 0.557881821 |
| ENSG00000137877 | 0.55766147 |
| ENSG00000183354 | 0.557205214 |
| ENSG00000188566 | 0.556871026 |
| ENSG00000241769 | 0.556751912 |
| ENSG00000176714 | 0.556380216 |
| ENSG00000075426 | 0.556128919 |
| ENSG00000133812 | 0.555176038 |
| ENSG00000105289 | 0.554407345 |
| ENSG00000153558 | 0.554212708 |
| ENSG00000168016 | 0.553889516 |

| | |
|---|---|
| ENSG00000054793 | 0.55346233 |
| ENSG00000068001 | 0.553203446 |
| ENSG00000153944 | 0.551320967 |
| ENSG00000236986 | 0.550941801 |
| ENSG00000178057 | 0.550794411 |
| ENSG00000260766 | 0.550533036 |
| ENSG00000226696 | 0.549456269 |
| ENSG00000243477 | 0.549142193 |
| ENSG00000120784 | 0.549043647 |
| ENSG00000020577 | 0.548852603 |
| ENSG00000167118 | 0.548531631 |
| ENSG00000269131 | 0.54806369 |
| ENSG00000137098 | 0.548049585 |
| ENSG00000235823 | 0.547868776 |
| ENSG00000060971 | 0.547547619 |
| ENSG00000165802 | 0.547381186 |
| ENSG00000130147 | 0.54717359 |
| ENSG00000106009 | 0.546953099 |
| ENSG00000226380 | 0.546504168 |
| ENSG00000047849 | 0.546418292 |
| ENSG00000225215 | 0.546400334 |
| ENSG00000157741 | 0.546334883 |
| ENSG00000268471 | 0.546088181 |
| ENSG00000167637 | 0.546000258 |
| ENSG00000155629 | 0.545613559 |
| ENSG00000196476 | 0.544655233 |
| ENSG00000158373 | 0.544494394 |
| ENSG00000227440 | 0.54433517 |
| ENSG00000062598 | 0.544082953 |
| ENSG00000213252 | 0.543654518 |
| ENSG00000148773 | 0.543533264 |
| ENSG00000160613 | 0.543422254 |
| ENSG00000174891 | 0.54325733 |
| ENSG00000128512 | 0.543029542 |
| ENSG00000140398 | 0.542882397 |
| ENSG00000126581 | 0.542594789 |
| ENSG00000167232 | 0.542482853 |
| ENSG00000235652 | 0.54245919 |
| ENSG00000174106 | 0.542319938 |
| ENSG00000104142 | 0.542240671 |

| | |
|---|---|
| ENSG00000130164 | 0.541969235 |
| ENSG00000260054 | 0.54170586 |
| ENSG00000163807 | 0.541588415 |
| ENSG00000272960 | 0.541157357 |
| ENSG00000273344 | 0.541024091 |
| ENSG00000067066 | 0.540380927 |
| ENSG00000270804 | 0.540303227 |
| ENSG00000242802 | 0.540136465 |
| ENSG00000083799 | 0.540071621 |
| ENSG00000270362 | 0.539967737 |
| ENSG00000100994 | 0.539937629 |
| ENSG00000154743 | 0.539814261 |
| ENSG00000137038 | 0.539757403 |
| ENSG00000223551 | 0.539525734 |
| ENSG00000107263 | 0.539443489 |
| ENSG00000245556 | 0.539210206 |
| ENSG00000151623 | 0.539206475 |
| ENSG00000032742 | 0.53919231 |
| ENSG00000166436 | 0.539154185 |
| ENSG00000099204 | 0.538798448 |
| ENSG00000260565 | 0.538784837 |
| ENSG00000197417 | 0.537947295 |
| ENSG00000125245 | 0.537276974 |
| ENSG00000157653 | 0.536486216 |
| ENSG00000143198 | 0.536142205 |
| ENSG00000167258 | 0.535039943 |
| ENSG00000197863 | 0.534680238 |
| ENSG00000167984 | 0.534651185 |
| ENSG00000186645 | 0.534608404 |
| ENSG00000095203 | 0.533886015 |
| ENSG00000176978 | 0.533823544 |
| ENSG00000170242 | 0.533442326 |
| ENSG00000185596 | 0.533183476 |
| ENSG00000188112 | 0.533007528 |
| ENSG00000196214 | 0.532852808 |
| ENSG00000179837 | 0.532836401 |
| ENSG00000149346 | 0.532533095 |
| ENSG00000218676 | 0.532359683 |
| ENSG00000148019 | 0.532239246 |
| ENSG00000263829 | 0.532014482 |

| | |
|---|---|
| ENSG00000176058 | 0.532013304 |
| ENSG00000198826 | 0.531655533 |
| ENSG00000185963 | 0.531354385 |
| ENSG00000257337 | 0.531330414 |
| ENSG00000157873 | 0.531310508 |
| ENSG00000134369 | 0.531260399 |
| ENSG00000196850 | 0.530353279 |
| ENSG00000198648 | 0.530261431 |
| ENSG00000180884 | 0.530173034 |
| ENSG00000227533 | 0.529841715 |
| ENSG00000159714 | 0.529436193 |
| ENSG00000145348 | 0.529307142 |
| ENSG00000080845 | 0.529305404 |
| ENSG00000240230 | 0.528825692 |
| ENSG00000122674 | 0.528235479 |
| ENSG00000272758 | 0.52788249 |
| ENSG00000227558 | 0.527604038 |
| ENSG00000184903 | 0.527538464 |
| ENSG00000111335 | 0.527492142 |
| ENSG00000197355 | 0.526822957 |
| ENSG00000168615 | 0.526449702 |
| ENSG00000125821 | 0.526412016 |
| ENSG00000196705 | 0.526202344 |
| ENSG00000259891 | 0.526059382 |
| ENSG00000260257 | 0.525911224 |
| ENSG00000110315 | 0.525474633 |
| ENSG00000196924 | 0.525308151 |
| ENSG00000163637 | 0.525226046 |
| ENSG00000160256 | 0.524926152 |
| ENSG00000106052 | 0.524809338 |
| ENSG00000158669 | 0.524287249 |
| ENSG00000072422 | 0.524200262 |
| ENSG00000270223 | 0.524179086 |
| ENSG00000134755 | 0.524168692 |
| ENSG00000081386 | 0.524153419 |
| ENSG00000271361 | 0.5241176 |
| ENSG00000125629 | 0.523734716 |
| ENSG00000235477 | 0.52360959 |
| ENSG00000120215 | 0.52353779 |
| ENSG00000145781 | 0.523476974 |

| | |
|---|---|
| ENSG00000106692 | 0.523215224 |
| ENSG00000075391 | 0.523134079 |
| ENSG00000125970 | 0.522949536 |
| ENSG00000161912 | 0.52282564 |
| ENSG00000107372 | 0.522567896 |
| ENSG00000170471 | 0.522492824 |
| ENSG00000188747 | 0.522481406 |
| ENSG00000123106 | 0.521933321 |
| ENSG00000169607 | 0.521757192 |
| ENSG00000204792 | 0.521705524 |
| ENSG00000079950 | 0.521300554 |
| ENSG00000188199 | 0.521260161 |
| ENSG00000127329 | 0.521144632 |
| ENSG00000262194 | 0.520777977 |
| ENSG00000263002 | 0.520570815 |
| ENSG00000089048 | 0.520087953 |
| ENSG00000059758 | 0.519407396 |
| ENSG00000106392 | 0.519050569 |
| ENSG00000134905 | 0.518504404 |
| ENSG00000136100 | 0.517705133 |
| ENSG00000144026 | 0.517684069 |
| ENSG00000132002 | 0.517583929 |
| ENSG00000127666 | 0.517443502 |
| ENSG00000134897 | 0.517352254 |
| ENSG00000213995 | 0.517305527 |
| ENSG00000271533 | 0.517209296 |
| ENSG00000273319 | 0.517017651 |
| ENSG00000156052 | 0.516604662 |
| ENSG00000035681 | 0.5160616 |
| ENSG00000133703 | 0.516034089 |
| ENSG00000068745 | 0.51602866 |
| ENSG00000111816 | 0.515998411 |
| ENSG00000232186 | 0.51560774 |
| ENSG00000143590 | 0.515045788 |
| ENSG00000080189 | 0.514721218 |
| ENSG00000267100 | 0.514443913 |
| ENSG00000122359 | 0.51418474 |
| ENSG00000204860 | 0.513981438 |
| ENSG00000198585 | 0.51366318 |
| ENSG00000204054 | 0.513601469 |

| | |
|---|---|
| ENSG00000153531 | 0.513301015 |
| ENSG00000261490 | 0.513142868 |
| ENSG00000130684 | 0.513087991 |
| ENSG00000232729 | 0.512728178 |
| ENSG00000124198 | 0.512650774 |
| ENSG00000126001 | 0.51240251 |
| ENSG00000187742 | 0.512039258 |
| ENSG00000146414 | 0.511971115 |
| ENSG00000242086 | 0.511712134 |
| ENSG00000165322 | 0.511677652 |
| ENSG00000197822 | 0.511389632 |
| ENSG00000146576 | 0.511165515 |
| ENSG00000135914 | 0.511147856 |
| ENSG00000102804 | 0.511110028 |
| ENSG00000147874 | 0.510575366 |
| ENSG00000155313 | 0.510470318 |
| ENSG00000239569 | 0.510450423 |
| ENSG00000253210 | 0.509983258 |
| ENSG00000126351 | 0.509934358 |
| ENSG00000013364 | 0.509933035 |
| ENSG00000108559 | 0.50983946 |
| ENSG00000130958 | 0.509439605 |
| ENSG00000083817 | 0.509318689 |
| ENSG00000242372 | 0.509024442 |
| ENSG00000175564 | 0.508897547 |
| ENSG00000120860 | 0.508837724 |
| ENSG00000011275 | 0.508578755 |
| ENSG00000112406 | 0.508374032 |
| ENSG00000125753 | 0.508101453 |
| ENSG00000106723 | 0.508045807 |
| ENSG00000163517 | 0.507631315 |
| ENSG00000188229 | 0.507142131 |
| ENSG00000184206 | 0.507106847 |
| ENSG00000224660 | 0.506513424 |
| ENSG00000180011 | 0.506050337 |
| ENSG00000178951 | 0.505936926 |
| ENSG00000198646 | 0.505806991 |
| ENSG00000149503 | 0.505271404 |
| ENSG00000085982 | 0.505095293 |
| ENSG00000117724 | 0.504672448 |

| | |
|---|---|
| ENSG00000070182 | 0.504508226 |
| ENSG00000148290 | 0.504437148 |
| ENSG00000119487 | 0.504434027 |
| ENSG00000198673 | 0.504429212 |
| ENSG00000230847 | 0.504315328 |
| ENSG00000187653 | 0.504304981 |
| ENSG00000012822 | 0.504179338 |
| ENSG00000156011 | 0.504034675 |
| ENSG00000116106 | 0.50379733 |
| ENSG00000102897 | 0.503605757 |
| ENSG00000170248 | 0.503424668 |
| ENSG00000106443 | 0.503119232 |
| ENSG00000155085 | 0.502876579 |
| ENSG00000272216 | 0.502857826 |
| ENSG00000120690 | 0.502659039 |
| ENSG00000167632 | 0.502452371 |
| ENSG00000130723 | 0.502377353 |
| ENSG00000138079 | 0.501994073 |
| ENSG00000233901 | 0.501914357 |
| ENSG00000165006 | 0.501882169 |
| ENSG00000101447 | 0.501317044 |
| ENSG00000129250 | 0.501116637 |
| ENSG00000160190 | 0.501055095 |
| ENSG00000152926 | 0.500832947 |
| ENSG00000213406 | 0.500828535 |
| ENSG00000168612 | 0.500584866 |
| ENSG00000235106 | 0.500506676 |
| ENSG00000121653 | 0.50043743 |
| ENSG00000242861 | 0.500300458 |
| ENSG00000147642 | 0.500251239 |
| ENSG00000068028 | 0.500235457 |
| ENSG00000124209 | 0.499917725 |
| ENSG00000139977 | 0.499872042 |
| ENSG00000127957 | 0.499740557 |
| ENSG00000004534 | 0.499642602 |
| ENSG00000175764 | 0.499568708 |
| ENSG00000233527 | 0.499501693 |
| ENSG00000139517 | 0.499476617 |
| ENSG00000130332 | 0.498206999 |
| ENSG00000177427 | 0.498124874 |

| | |
|---|---|
| ENSG00000145439 | 0.497704911 |
| ENSG00000240288 | 0.497639151 |
| ENSG00000163930 | 0.497408021 |
| ENSG00000182718 | 0.496945681 |
| ENSG00000233903 | 0.496560251 |
| ENSG00000101400 | 0.496414419 |
| ENSG00000133401 | 0.496124737 |
| ENSG00000267742 | 0.496077725 |
| ENSG00000148334 | 0.49544811 |
| ENSG00000181004 | 0.495340726 |
| ENSG00000105926 | 0.495243085 |
| ENSG00000158286 | 0.494648198 |
| ENSG00000161642 | 0.494594868 |
| ENSG00000179588 | 0.493900513 |
| ENSG00000063176 | 0.493884763 |
| ENSG00000169047 | 0.493705529 |
| ENSG00000196418 | 0.493589136 |
| ENSG00000197043 | 0.493446032 |
| ENSG00000075239 | 0.49302374 |
| ENSG00000166833 | 0.492799664 |
| ENSG00000229186 | 0.492722644 |
| ENSG00000148468 | 0.492038991 |
| ENSG00000089177 | 0.491492664 |
| ENSG00000116586 | 0.491047408 |
| ENSG00000103148 | 0.490699534 |
| ENSG00000234028 | 0.490349968 |
| ENSG00000225135 | 0.490257294 |
| ENSG00000182957 | 0.489887809 |
| ENSG00000225138 | 0.489331377 |
| ENSG00000175873 | 0.489178096 |
| ENSG00000105339 | 0.48897596 |
| ENSG00000236526 | 0.488519037 |
| ENSG00000164631 | 0.488174089 |
| ENSG00000144476 | 0.487819134 |
| ENSG00000226137 | 0.487321037 |
| ENSG00000131473 | 0.487316218 |
| ENSG00000174374 | 0.487141095 |
| ENSG00000198783 | 0.486892025 |
| ENSG00000127081 | 0.486754867 |
| ENSG00000148814 | 0.486737162 |

| | |
|---|---|
| ENSG00000140853 | 0.486575372 |
| ENSG00000119383 | 0.48592307 |
| ENSG00000211455 | 0.485590441 |
| ENSG00000124831 | 0.485555778 |
| ENSG00000127870 | 0.484940396 |
| ENSG00000180881 | 0.484875387 |
| ENSG00000186322 | 0.484590308 |
| ENSG00000148180 | 0.483859082 |
| ENSG00000174032 | 0.483837925 |
| ENSG00000141252 | 0.483723525 |
| ENSG00000261485 | 0.483696032 |
| ENSG00000138593 | 0.483678031 |
| ENSG00000231991 | 0.483428708 |
| ENSG00000152061 | 0.48330411 |
| ENSG00000176472 | 0.483179256 |
| ENSG00000099849 | 0.483118495 |
| ENSG00000182247 | 0.482521139 |
| ENSG00000088888 | 0.482177354 |
| ENSG00000214021 | 0.482108101 |
| ENSG00000138246 | 0.482048539 |
| ENSG00000148297 | 0.481327408 |
| ENSG00000158711 | 0.48125644 |
| ENSG00000136856 | 0.481214299 |
| ENSG00000136824 | 0.481178199 |
| ENSG00000203993 | 0.481140577 |
| ENSG00000183283 | 0.48111661 |
| ENSG00000104883 | 0.480897878 |
| ENSG00000086102 | 0.480623908 |
| ENSG00000141570 | 0.480602576 |
| ENSG00000142634 | 0.480349746 |
| ENSG00000029153 | 0.480317292 |
| ENSG00000136854 | 0.480205021 |
| ENSG00000256223 | 0.480189048 |
| ENSG00000106397 | 0.479939206 |
| ENSG00000181322 | 0.479839466 |
| ENSG00000162755 | 0.479823787 |
| ENSG00000224959 | 0.479700721 |
| ENSG00000139323 | 0.479522778 |
| ENSG00000164292 | 0.479230439 |
| ENSG00000196151 | 0.479061319 |

| | |
|---|---|
| ENSG00000114742 | 0.478870273 |
| ENSG00000101040 | 0.478647697 |
| ENSG00000139146 | 0.478441889 |
| ENSG00000105717 | 0.478017802 |
| ENSG00000138642 | 0.477953686 |
| ENSG00000164880 | 0.477498521 |
| ENSG00000163399 | 0.47745632 |
| ENSG00000272079 | 0.477034221 |
| ENSG00000142235 | 0.476987755 |
| ENSG00000167716 | 0.476636912 |
| ENSG00000197372 | 0.476516594 |
| ENSG00000064607 | 0.476421034 |
| ENSG00000185305 | 0.47573347 |
| ENSG00000114302 | 0.475695352 |
| ENSG00000170876 | 0.475515995 |
| ENSG00000041880 | 0.475356285 |
| ENSG00000270346 | 0.475313131 |
| ENSG00000064666 | 0.474935733 |
| ENSG00000130517 | 0.474876813 |
| ENSG00000111790 | 0.474865866 |
| ENSG00000118898 | 0.474836362 |
| ENSG00000230787 | 0.474536356 |
| ENSG00000183826 | 0.474435831 |
| ENSG00000144468 | 0.474301534 |
| ENSG00000125686 | 0.474145136 |
| ENSG00000139263 | 0.474048708 |
| ENSG00000139626 | 0.473711991 |
| ENSG00000010318 | 0.473423073 |
| ENSG00000132604 | 0.473409293 |
| ENSG00000040531 | 0.473403126 |
| ENSG00000114383 | 0.473322185 |
| ENSG00000160360 | 0.473102703 |
| ENSG00000230358 | 0.472676661 |
| ENSG00000182150 | 0.472564962 |
| ENSG00000131773 | 0.472422069 |
| ENSG00000170092 | 0.47223267 |
| ENSG00000177602 | 0.472196728 |
| ENSG00000169914 | 0.471897052 |
| ENSG00000181090 | 0.471784018 |
| ENSG00000103222 | 0.47134378 |

| | |
|---|---|
| ENSG00000150457 | 0.471286882 |
| ENSG00000169981 | 0.470702893 |
| ENSG00000225190 | 0.470467006 |
| ENSG00000159685 | 0.470463365 |
| ENSG00000112378 | 0.469900106 |
| ENSG00000119509 | 0.469449793 |
| ENSG00000186654 | 0.469371734 |
| ENSG00000113504 | 0.469116241 |
| ENSG00000196642 | 0.468347268 |
| ENSG00000139793 | 0.468145772 |
| ENSG00000177469 | 0.46813446 |
| ENSG00000248008 | 0.46761776 |
| ENSG00000113048 | 0.467307051 |
| ENSG00000257489 | 0.467280914 |
| ENSG00000132792 | 0.467023659 |
| ENSG00000169155 | 0.466968549 |
| ENSG00000146006 | 0.466611984 |
| ENSG00000224226 | 0.466570235 |
| ENSG00000198373 | 0.466538806 |
| ENSG00000188342 | 0.466518087 |
| ENSG00000141854 | 0.46632993 |
| ENSG00000243302 | 0.465625268 |
| ENSG00000141084 | 0.465124775 |
| ENSG00000163788 | 0.464669353 |
| ENSG00000105443 | 0.463798317 |
| ENSG00000261799 | 0.4633773 |
| ENSG00000147601 | 0.463357192 |
| ENSG00000164105 | 0.463109236 |
| ENSG00000131069 | 0.462848548 |
| ENSG00000228327 | 0.46283073 |
| ENSG00000159917 | 0.462564379 |
| ENSG00000166987 | 0.462149186 |
| ENSG00000104866 | 0.462031426 |
| ENSG00000204794 | 0.461724869 |
| ENSG00000165525 | 0.461193391 |
| ENSG00000163812 | 0.4609947 |
| ENSG00000139437 | 0.460575777 |
| ENSG00000051341 | 0.460525115 |
| ENSG00000134899 | 0.460097929 |
| ENSG00000107968 | 0.460087256 |

| | |
|---|---|
| ENSG00000039650 | 0.459937285 |
| ENSG00000071189 | 0.45973208 |
| ENSG00000105136 | 0.459326732 |
| ENSG00000177479 | 0.459301489 |
| ENSG00000188825 | 0.458762521 |
| ENSG00000106049 | 0.458512735 |
| ENSG00000146376 | 0.458460863 |
| ENSG00000106346 | 0.458235936 |
| ENSG00000073350 | 0.458044335 |
| ENSG00000056972 | 0.457544074 |
| ENSG00000123989 | 0.457492303 |
| ENSG00000227344 | 0.457472416 |
| ENSG00000168036 | 0.456984323 |
| ENSG00000163808 | 0.456845541 |
| ENSG00000259086 | 0.456531432 |
| ENSG00000176619 | 0.456222984 |
| ENSG00000256683 | 0.455649809 |
| ENSG00000196116 | 0.45519002 |
| ENSG00000231742 | 0.454432415 |
| ENSG00000129810 | 0.454377684 |
| ENSG00000105287 | 0.454374288 |
| ENSG00000142002 | 0.454151727 |
| ENSG00000188215 | 0.454108007 |
| ENSG00000127191 | 0.45369607 |
| ENSG00000100036 | 0.453366441 |
| ENSG00000196670 | 0.453206785 |
| ENSG00000174238 | 0.453196134 |
| ENSG00000260336 | 0.453162551 |
| ENSG00000197776 | 0.453152227 |
| ENSG00000117868 | 0.45277784 |
| ENSG00000146540 | 0.452696032 |
| ENSG00000251247 | 0.452508393 |
| ENSG00000183255 | 0.452418243 |
| ENSG00000014216 | 0.451899288 |
| ENSG00000188388 | 0.451279121 |
| ENSG00000160199 | 0.450889965 |
| ENSG00000137812 | 0.450806411 |
| ENSG00000149657 | 0.450247927 |
| ENSG00000233822 | 0.450071501 |
| ENSG00000105053 | 0.449992483 |

| | |
|---|---|
| ENSG00000170365 | 0.449799633 |
| ENSG00000164535 | 0.449743603 |
| ENSG00000144134 | 0.449508869 |
| ENSG00000072818 | 0.448919444 |
| ENSG00000110400 | 0.448859817 |
| ENSG00000198496 | 0.448833423 |
| ENSG00000124091 | 0.448503111 |
| ENSG00000248923 | 0.447949672 |
| ENSG00000269979 | 0.44753489 |
| ENSG00000125482 | 0.447503269 |
| ENSG00000114166 | 0.447488697 |
| ENSG00000261338 | 0.447412886 |
| ENSG00000186088 | 0.447273044 |
| ENSG00000198887 | 0.447210368 |
| ENSG00000165527 | 0.446773009 |
| ENSG00000101363 | 0.446746855 |
| ENSG00000111726 | 0.446647744 |
| ENSG00000269501 | 0.446632476 |
| ENSG00000189007 | 0.446225605 |
| ENSG00000225373 | 0.44608636 |
| ENSG00000130338 | 0.446026988 |
| ENSG00000170871 | 0.445609927 |
| ENSG00000158246 | 0.445312663 |
| ENSG00000181638 | 0.444865085 |
| ENSG00000136819 | 0.444844099 |
| ENSG00000186918 | 0.444810062 |
| ENSG00000135040 | 0.444798953 |
| ENSG00000145780 | 0.444778475 |
| ENSG00000103187 | 0.443843481 |
| ENSG00000124224 | 0.443602739 |
| ENSG00000180938 | 0.443445226 |
| ENSG00000091073 | 0.443037403 |
| ENSG00000138669 | 0.442966891 |
| ENSG00000013573 | 0.442631131 |
| ENSG00000107262 | 0.442586001 |
| ENSG00000119522 | 0.442531202 |
| ENSG00000107362 | 0.441752618 |
| ENSG00000055609 | 0.441597855 |
| ENSG00000107338 | 0.441253305 |
| ENSG00000100300 | 0.441196169 |

| | |
|---|---|
| ENSG00000119771 | 0.441070426 |
| ENSG00000205508 | 0.440508062 |
| ENSG00000197008 | 0.439843141 |
| ENSG00000148358 | 0.439808985 |
| ENSG00000273015 | 0.439616244 |
| ENSG00000260778 | 0.439566237 |
| ENSG00000236060 | 0.439299246 |
| ENSG00000198917 | 0.439235143 |
| ENSG00000178209 | 0.438951672 |
| ENSG00000011376 | 0.438671068 |
| ENSG00000167608 | 0.438549513 |
| ENSG00000096968 | 0.438171808 |
| ENSG00000160336 | 0.438064819 |
| ENSG00000123975 | 0.437991933 |
| ENSG00000155827 | 0.437977632 |
| ENSG00000143190 | 0.437790283 |
| ENSG00000176563 | 0.437607081 |
| ENSG00000261474 | 0.437533447 |
| ENSG00000023287 | 0.437234738 |
| ENSG00000229180 | 0.437199677 |
| ENSG00000249936 | 0.436651553 |
| ENSG00000137076 | 0.43647799 |
| ENSG00000213928 | 0.436441691 |
| ENSG00000226752 | 0.435807281 |
| ENSG00000165807 | 0.43544363 |
| ENSG00000172113 | 0.435071274 |
| ENSG00000266563 | 0.434808612 |
| ENSG00000089639 | 0.434451476 |
| ENSG00000149084 | 0.434079925 |
| ENSG00000196967 | 0.433824316 |
| ENSG00000181220 | 0.433727279 |
| ENSG00000101052 | 0.433723536 |
| ENSG00000104331 | 0.433390521 |
| ENSG00000145685 | 0.43336156 |
| ENSG00000176658 | 0.433092262 |
| ENSG00000174365 | 0.432863468 |
| ENSG00000067082 | 0.432857661 |
| ENSG00000227388 | 0.432827123 |
| ENSG00000119318 | 0.432503082 |
| ENSG00000167671 | 0.432119315 |

| | |
|---|---|
| ENSG00000167840 | 0.43205901 |
| ENSG00000179456 | 0.43203267 |
| ENSG00000205580 | 0.431786592 |
| ENSG00000135506 | 0.431714455 |
| ENSG00000102554 | 0.431675057 |
| ENSG00000266930 | 0.431429897 |
| ENSG00000172292 | 0.431418496 |
| ENSG00000156697 | 0.431399439 |
| ENSG00000100599 | 0.431098511 |
| ENSG00000034152 | 0.431066856 |
| ENSG00000115183 | 0.430702996 |
| ENSG00000273117 | 0.430249438 |
| ENSG00000221817 | 0.430208987 |
| ENSG00000106211 | 0.430154989 |
| ENSG00000181894 | 0.430111755 |
| ENSG00000166526 | 0.42994243 |
| ENSG00000247903 | 0.42957018 |
| ENSG00000260034 | 0.42920034 |
| ENSG00000235423 | 0.428892561 |
| ENSG00000163590 | 0.428522078 |
| ENSG00000143933 | 0.427113043 |
| ENSG00000272782 | 0.426762588 |
| ENSG00000247796 | 0.426706019 |
| ENSG00000100504 | 0.426621511 |
| ENSG00000198863 | 0.426364744 |
| ENSG00000229645 | 0.425716726 |
| ENSG00000257279 | 0.425239354 |
| ENSG00000230373 | 0.425222266 |
| ENSG00000135269 | 0.425178106 |
| ENSG00000177731 | 0.425172124 |
| ENSG00000177225 | 0.424914037 |
| ENSG00000110619 | 0.4247577 |
| ENSG00000181467 | 0.424430519 |
| ENSG00000198464 | 0.424413421 |
| ENSG00000149600 | 0.424389636 |
| ENSG00000203667 | 0.424343324 |
| ENSG00000105402 | 0.424146834 |
| ENSG00000139083 | 0.424030531 |
| ENSG00000163939 | 0.423387032 |
| ENSG00000180423 | 0.423267814 |

| | |
|---|---|
| ENSG00000124181 | 0.423260915 |
| ENSG00000121417 | 0.422378291 |
| ENSG00000188554 | 0.421655571 |
| ENSG00000176248 | 0.421598738 |
| ENSG00000103342 | 0.421526134 |
| ENSG00000213918 | 0.42120305 |
| ENSG00000234769 | 0.421186477 |
| ENSG00000144824 | 0.421020968 |
| ENSG00000107341 | 0.420569604 |
| ENSG00000146707 | 0.42038556 |
| ENSG00000272091 | 0.420237962 |
| ENSG00000196696 | 0.420063618 |
| ENSG00000214188 | 0.419650916 |
| ENSG00000188152 | 0.419302662 |
| ENSG00000188191 | 0.419223243 |
| ENSG00000175220 | 0.419093 |
| ENSG00000171097 | 0.418822695 |
| ENSG00000162600 | 0.418684408 |
| ENSG00000101997 | 0.418318531 |
| ENSG00000104946 | 0.418302504 |
| ENSG00000174194 | 0.418297169 |
| ENSG00000179941 | 0.418241215 |
| ENSG00000144445 | 0.418189259 |
| ENSG00000135074 | 0.418182886 |
| ENSG00000166348 | 0.418156872 |
| ENSG00000172986 | 0.417924964 |
| ENSG00000226361 | 0.417475307 |
| ENSG00000133704 | 0.417382133 |
| ENSG00000080819 | 0.41720519 |
| ENSG00000106992 | 0.416961775 |
| ENSG00000230797 | 0.416952095 |
| ENSG00000145819 | 0.416874831 |
| ENSG00000112679 | 0.41686337 |
| ENSG00000124160 | 0.416796767 |
| ENSG00000178764 | 0.415945766 |
| ENSG00000174171 | 0.415099904 |
| ENSG00000163659 | 0.414970194 |
| ENSG00000078699 | 0.414878878 |
| ENSG00000046651 | 0.414797441 |
| ENSG00000167994 | 0.414612315 |

| | |
|---|---|
| ENSG00000151164 | 0.414594916 |
| ENSG00000101493 | 0.414450771 |
| ENSG00000124151 | 0.414358271 |
| ENSG00000150773 | 0.414141764 |
| ENSG00000103479 | 0.414045323 |
| ENSG00000119523 | 0.413740666 |
| ENSG00000179598 | 0.413377845 |
| ENSG00000267288 | 0.413273285 |
| ENSG00000164081 | 0.413228948 |
| ENSG00000272947 | 0.413175734 |
| ENSG00000133313 | 0.4130757 |
| ENSG00000166224 | 0.413005057 |
| ENSG00000182179 | 0.412586714 |
| ENSG00000103111 | 0.412532963 |
| ENSG00000125827 | 0.412390832 |
| ENSG00000125648 | 0.412260242 |
| ENSG00000113645 | 0.412230908 |
| ENSG00000175283 | 0.412226531 |
| ENSG00000267242 | 0.412041008 |
| ENSG00000234420 | 0.411990579 |
| ENSG00000006652 | 0.41188585 |
| ENSG00000204104 | 0.411826478 |
| ENSG00000262943 | 0.411760365 |
| ENSG00000227232 | 0.411587223 |
| ENSG00000170545 | 0.411282684 |
| ENSG00000146757 | 0.411191664 |
| ENSG00000165282 | 0.411107806 |
| ENSG00000171100 | 0.410873716 |
| ENSG00000142528 | 0.410719326 |
| ENSG00000160193 | 0.410276087 |
| ENSG00000186480 | 0.410199684 |
| ENSG00000160818 | 0.410062229 |
| ENSG00000159842 | 0.40968695 |
| ENSG00000048405 | 0.409604153 |
| ENSG00000131375 | 0.409551183 |
| ENSG00000265194 | 0.409411984 |
| ENSG00000170439 | 0.409145814 |
| ENSG00000259480 | 0.40897904 |
| ENSG00000229097 | 0.408932017 |
| ENSG00000136807 | 0.40870837 |

| | |
|---|---|
| ENSG00000159882 | 0.408567327 |
| ENSG00000147439 | 0.408436923 |
| ENSG00000181896 | 0.408291689 |
| ENSG00000101452 | 0.408269746 |
| ENSG00000167113 | 0.407267873 |
| ENSG00000100503 | 0.406956388 |
| ENSG00000185829 | 0.406890578 |
| ENSG00000136870 | 0.406643171 |
| ENSG00000118503 | 0.406550332 |
| ENSG00000239305 | 0.406110039 |
| ENSG00000037897 | 0.405984243 |
| ENSG00000204116 | 0.405886852 |
| ENSG00000170425 | 0.405709254 |
| ENSG00000271430 | 0.405473945 |
| ENSG00000247271 | 0.405472652 |
| ENSG00000266469 | 0.405205543 |
| ENSG00000106993 | 0.405162167 |
| ENSG00000160007 | 0.40499583 |
| ENSG00000107020 | 0.404663466 |
| ENSG00000128656 | 0.404659545 |
| ENSG00000176148 | 0.403443999 |
| ENSG00000168237 | 0.403307451 |
| ENSG00000221962 | 0.403198952 |
| ENSG00000117335 | 0.403111427 |
| ENSG00000165138 | 0.402827341 |
| ENSG00000071564 | 0.402795085 |
| ENSG00000076351 | 0.402587706 |
| ENSG00000073584 | 0.402560645 |
| ENSG00000172660 | 0.402317765 |
| ENSG00000197930 | 0.40214619 |
| ENSG00000249839 | 0.402111356 |
| ENSG00000188033 | 0.402028617 |
| ENSG00000268869 | 0.401768084 |
| ENSG00000253106 | 0.401415884 |
| ENSG00000186472 | 0.401395753 |
| ENSG00000102780 | 0.401382513 |
| ENSG00000132825 | 0.401355102 |
| ENSG00000088387 | 0.401300729 |
| ENSG00000095319 | 0.401281772 |
| ENSG00000079974 | 0.401244839 |

| | |
|---|---|
| ENSG00000178234 | 0.401033939 |
| ENSG00000104964 | 0.400764034 |
| ENSG00000137101 | 0.400631784 |
| ENSG00000236144 | 0.400598651 |
| ENSG00000122565 | 0.400579631 |
| ENSG00000166046 | 0.400552153 |
| ENSG00000172785 | 0.400387411 |
| ENSG00000204611 | 0.400177486 |
| ENSG00000112787 | 0.399850451 |
| ENSG00000167468 | 0.399636618 |
| ENSG00000108861 | 0.399561534 |
| ENSG00000114904 | 0.399345269 |
| ENSG00000164068 | 0.39915562 |
| ENSG00000163644 | 0.398608621 |
| ENSG00000125812 | 0.398542802 |
| ENSG00000228696 | 0.398185344 |
| ENSG00000130589 | 0.397545622 |
| ENSG00000139233 | 0.397434881 |
| ENSG00000148737 | 0.397045718 |
| ENSG00000173295 | 0.396590431 |
| ENSG00000133216 | 0.396477812 |
| ENSG00000126653 | 0.396393331 |
| ENSG00000134318 | 0.396382603 |
| ENSG00000232956 | 0.395752658 |
| ENSG00000221883 | 0.395751875 |
| ENSG00000121680 | 0.39570339 |
| ENSG00000136811 | 0.39563664 |
| ENSG00000136932 | 0.395424662 |
| ENSG00000260917 | 0.395368355 |
| ENSG00000179630 | 0.395088485 |
| ENSG00000126461 | 0.394960827 |
| ENSG00000229539 | 0.39473089 |
| ENSG00000196371 | 0.394648973 |
| ENSG00000126062 | 0.39456881 |
| ENSG00000107036 | 0.393480572 |
| ENSG00000212998 | 0.393372076 |
| ENSG00000165813 | 0.393317966 |
| ENSG00000171970 | 0.393300613 |
| ENSG00000160191 | 0.393021967 |
| ENSG00000148384 | 0.392845857 |

| | |
|---|---|
| ENSG00000124214 | 0.392679591 |
| ENSG00000164465 | 0.392678156 |
| ENSG00000196152 | 0.392643163 |
| ENSG00000187609 | 0.392164938 |
| ENSG00000234327 | 0.392087934 |
| ENSG00000229728 | 0.391719748 |
| ENSG00000119335 | 0.391649259 |
| ENSG00000198816 | 0.391611439 |
| ENSG00000064102 | 0.391474894 |
| ENSG00000060491 | 0.391461803 |
| ENSG00000064601 | 0.391042209 |
| ENSG00000140941 | 0.390826326 |
| ENSG00000164818 | 0.39047159 |
| ENSG00000084731 | 0.389910951 |
| ENSG00000239467 | 0.389679513 |
| ENSG00000174282 | 0.388747848 |
| ENSG00000215102 | 0.388594671 |
| ENSG00000129667 | 0.388592185 |
| ENSG00000159461 | 0.388456109 |
| ENSG00000135473 | 0.388157601 |
| ENSG00000125375 | 0.387762047 |
| ENSG00000197702 | 0.387599416 |
| ENSG00000165689 | 0.387400604 |
| ENSG00000163516 | 0.387344066 |
| ENSG00000174292 | 0.387125115 |
| ENSG00000080839 | 0.387072454 |
| ENSG00000166321 | 0.386706539 |
| ENSG00000159069 | 0.386479473 |
| ENSG00000229833 | 0.386042055 |
| ENSG00000157107 | 0.38591402 |
| ENSG00000112584 | 0.385293153 |
| ENSG00000009413 | 0.38528211 |
| ENSG00000197461 | 0.385132097 |
| ENSG00000151148 | 0.385091058 |
| ENSG00000127804 | 0.384970966 |
| ENSG00000213144 | 0.384940301 |
| ENSG00000135698 | 0.384366893 |
| ENSG00000164916 | 0.384347771 |
| ENSG00000132286 | 0.384312675 |
| ENSG00000133275 | 0.384286082 |

| | |
|---|---|
| ENSG00000144485 | 0.384054991 |
| ENSG00000156675 | 0.384007723 |
| ENSG00000175866 | 0.383835505 |
| ENSG00000086232 | 0.383687187 |
| ENSG00000228638 | 0.383636152 |
| ENSG00000167112 | 0.382775092 |
| ENSG00000272669 | 0.382635274 |
| ENSG00000165699 | 0.382546412 |
| ENSG00000074755 | 0.382432726 |
| ENSG00000228300 | 0.382374867 |
| ENSG00000168517 | 0.382127288 |
| ENSG00000101473 | 0.381781514 |
| ENSG00000137145 | 0.381595531 |
| ENSG00000267023 | 0.381526948 |
| ENSG00000197961 | 0.381408503 |
| ENSG00000088812 | 0.381256688 |
| ENSG00000151092 | 0.380254621 |
| ENSG00000188760 | 0.379634083 |
| ENSG00000129197 | 0.378851821 |
| ENSG00000148343 | 0.37876579 |
| ENSG00000119950 | 0.378340662 |
| ENSG00000161956 | 0.377749628 |
| ENSG00000211456 | 0.377121122 |
| ENSG00000141582 | 0.377062463 |
| ENSG00000185621 | 0.37651415 |
| ENSG00000205707 | 0.37607466 |
| ENSG00000123810 | 0.375887826 |
| ENSG00000112079 | 0.375679373 |
| ENSG00000130962 | 0.375036938 |
| ENSG00000166261 | 0.374727009 |
| ENSG00000237857 | 0.374688455 |
| ENSG00000171681 | 0.374203585 |
| ENSG00000197081 | 0.37413846 |
| ENSG00000154642 | 0.374032932 |
| ENSG00000188559 | 0.373887139 |
| ENSG00000198301 | 0.373651242 |
| ENSG00000136908 | 0.373266546 |
| ENSG00000111331 | 0.373110437 |
| ENSG00000107077 | 0.372684381 |
| ENSG00000164877 | 0.37268381 |

| | |
|---|---|
| ENSG00000164976 | 0.372404651 |
| ENSG00000196081 | 0.372165511 |
| ENSG00000110880 | 0.372017278 |
| ENSG00000272003 | 0.371748806 |
| ENSG00000135052 | 0.371606869 |
| ENSG00000157224 | 0.370909882 |
| ENSG00000107819 | 0.370497685 |
| ENSG00000101407 | 0.370346176 |
| ENSG00000231312 | 0.37026761 |
| ENSG00000130340 | 0.369882021 |
| ENSG00000032444 | 0.369769037 |
| ENSG00000130940 | 0.36972876 |
| ENSG00000215817 | 0.369637193 |
| ENSG00000105793 | 0.369148284 |
| ENSG00000126368 | 0.368390523 |
| ENSG00000167065 | 0.367914515 |
| ENSG00000123213 | 0.367913727 |
| ENSG00000165487 | 0.367738473 |
| ENSG00000148331 | 0.367514222 |
| ENSG00000148120 | 0.367503018 |
| ENSG00000126003 | 0.366138445 |
| ENSG00000110651 | 0.36581452 |
| ENSG00000175203 | 0.365782178 |
| ENSG00000248932 | 0.365746303 |
| ENSG00000152642 | 0.365399311 |
| ENSG00000271870 | 0.365293175 |
| ENSG00000088298 | 0.364821117 |
| ENSG00000145041 | 0.364727775 |
| ENSG00000114767 | 0.364365713 |
| ENSG00000188542 | 0.364278168 |
| ENSG00000109576 | 0.363621298 |
| ENSG00000132109 | 0.363540789 |
| ENSG00000184752 | 0.363480047 |
| ENSG00000136897 | 0.363372395 |
| ENSG00000091527 | 0.362502525 |
| ENSG00000050426 | 0.362401408 |
| ENSG00000070476 | 0.362194951 |
| ENSG00000198876 | 0.362121354 |
| ENSG00000139651 | 0.361830172 |
| ENSG00000112282 | 0.361741233 |

| | |
|---|---|
| ENSG00000260261 | 0.361390674 |
| ENSG00000175063 | 0.360924615 |
| ENSG00000072310 | 0.360898359 |
| ENSG00000164548 | 0.360852069 |
| ENSG00000214706 | 0.360798246 |
| ENSG00000116539 | 0.360782698 |
| ENSG00000105447 | 0.360465116 |
| ENSG00000125633 | 0.360303976 |
| ENSG00000075914 | 0.36014813 |
| ENSG00000184787 | 0.360042236 |
| ENSG00000131368 | 0.359929128 |
| ENSG00000035862 | 0.359774743 |
| ENSG00000076201 | 0.359764509 |
| ENSG00000138386 | 0.359621069 |
| ENSG00000103126 | 0.359425247 |
| ENSG00000085377 | 0.359403889 |
| ENSG00000154144 | 0.359306009 |
| ENSG00000088325 | 0.357947385 |
| ENSG00000136144 | 0.357848273 |
| ENSG00000238165 | 0.357775539 |
| ENSG00000089063 | 0.357668442 |
| ENSG00000113522 | 0.357168877 |
| ENSG00000081026 | 0.356614527 |
| ENSG00000146587 | 0.356478831 |
| ENSG00000120688 | 0.356413215 |
| ENSG00000183337 | 0.356237155 |
| ENSG00000240489 | 0.356228439 |
| ENSG00000169871 | 0.356151523 |
| ENSG00000174912 | 0.356067345 |
| ENSG00000068120 | 0.355893395 |
| ENSG00000003147 | 0.355827957 |
| ENSG00000128578 | 0.355598897 |
| ENSG00000016864 | 0.354852553 |
| ENSG00000177697 | 0.354764126 |
| ENSG00000122034 | 0.354417796 |
| ENSG00000100982 | 0.354349934 |
| ENSG00000119392 | 0.354204772 |
| ENSG00000163171 | 0.353852102 |
| ENSG00000107223 | 0.353648903 |
| ENSG00000081307 | 0.353569527 |

| | |
|---|---|
| ENSG00000126858 | 0.35346418 |
| ENSG00000142599 | 0.353195774 |
| ENSG00000122512 | 0.352606342 |
| ENSG00000168255 | 0.352085755 |
| ENSG00000127914 | 0.351908317 |
| ENSG00000136861 | 0.350947687 |
| ENSG00000115649 | 0.350837432 |
| ENSG00000183161 | 0.350489909 |
| ENSG00000127328 | 0.350478315 |
| ENSG00000122033 | 0.350375024 |
| ENSG00000106477 | 0.350254644 |
| ENSG00000067248 | 0.350168394 |
| ENSG00000160201 | 0.349646207 |
| ENSG00000167110 | 0.349419094 |
| ENSG00000120868 | 0.34936996 |
| ENSG00000177119 | 0.348253682 |
| ENSG00000123159 | 0.347873418 |
| ENSG00000188986 | 0.347680684 |
| ENSG00000204842 | 0.347644303 |
| ENSG00000114529 | 0.347612284 |
| ENSG00000259623 | 0.347497415 |
| ENSG00000198920 | 0.347094478 |
| ENSG00000217128 | 0.346849308 |
| ENSG00000160799 | 0.346791422 |
| ENSG00000106780 | 0.346663484 |
| ENSG00000270102 | 0.345684324 |
| ENSG00000176624 | 0.345684218 |
| ENSG00000033627 | 0.345666726 |
| ENSG00000188994 | 0.345519608 |
| ENSG00000131051 | 0.345387647 |
| ENSG00000188647 | 0.345021027 |
| ENSG00000061938 | 0.345017618 |
| ENSG00000172939 | 0.344611414 |
| ENSG00000130699 | 0.344588798 |
| ENSG00000087448 | 0.34455175 |
| ENSG00000111670 | 0.344294488 |
| ENSG00000100991 | 0.344187457 |
| ENSG00000174306 | 0.343459097 |
| ENSG00000173209 | 0.343034603 |
| ENSG00000198131 | 0.342980099 |

| | |
|---|---|
| ENSG00000173276 | 0.342955509 |
| ENSG00000165490 | 0.342834989 |
| ENSG00000164978 | 0.342803109 |
| ENSG00000220583 | 0.342799801 |
| ENSG00000078902 | 0.342620496 |
| ENSG00000148399 | 0.342397037 |
| ENSG00000022567 | 0.342311578 |
| ENSG00000147144 | 0.34202662 |
| ENSG00000164610 | 0.341768846 |
| ENSG00000198839 | 0.341620972 |
| ENSG00000070269 | 0.341494588 |
| ENSG00000113812 | 0.340689498 |
| ENSG00000121057 | 0.340620039 |
| ENSG00000120451 | 0.34019412 |
| ENSG00000122515 | 0.340153797 |
| ENSG00000124145 | 0.33967697 |
| ENSG00000177463 | 0.339273666 |
| ENSG00000109686 | 0.338483724 |
| ENSG00000075651 | 0.338051422 |
| ENSG00000106701 | 0.337033932 |
| ENSG00000075785 | 0.336715299 |
| ENSG00000138764 | 0.336378565 |
| ENSG00000110721 | 0.335895551 |
| ENSG00000117650 | 0.334918834 |
| ENSG00000168297 | 0.334669054 |
| ENSG00000146833 | 0.334636842 |
| ENSG00000003393 | 0.334234755 |
| ENSG00000198730 | 0.33415511 |
| ENSG00000136238 | 0.333838997 |
| ENSG00000075856 | 0.333741753 |
| ENSG00000083168 | 0.333417 |
| ENSG00000125834 | 0.333358781 |
| ENSG00000106244 | 0.332983581 |
| ENSG00000134352 | 0.332747247 |
| ENSG00000107201 | 0.332315657 |
| ENSG00000015171 | 0.332234503 |
| ENSG00000073008 | 0.331907582 |
| ENSG00000118518 | 0.331622946 |
| ENSG00000106263 | 0.331533286 |
| ENSG00000068024 | 0.331422666 |

| | |
|---|---|
| ENSG00000167202 | 0.331157402 |
| ENSG00000132275 | 0.33100719 |
| ENSG00000172315 | 0.330969184 |
| ENSG00000163938 | 0.33030221 |
| ENSG00000100422 | 0.330180114 |
| ENSG00000090097 | 0.329996053 |
| ENSG00000131941 | 0.329693247 |
| ENSG00000165233 | 0.329369348 |
| ENSG00000131043 | 0.329241356 |
| ENSG00000080608 | 0.328342833 |
| ENSG00000166337 | 0.327999044 |
| ENSG00000206573 | 0.327946128 |
| ENSG00000132361 | 0.327541629 |
| ENSG00000186577 | 0.327452398 |
| ENSG00000136891 | 0.32684428 |
| ENSG00000108510 | 0.326412972 |
| ENSG00000143315 | 0.326096509 |
| ENSG00000102317 | 0.325892505 |
| ENSG00000074356 | 0.325892288 |
| ENSG00000175893 | 0.324849049 |
| ENSG00000268205 | 0.324679526 |
| ENSG00000187866 | 0.324011012 |
| ENSG00000197969 | 0.323562248 |
| ENSG00000167470 | 0.323425621 |
| ENSG00000075407 | 0.32334922 |
| ENSG00000149716 | 0.322955462 |
| ENSG00000196683 | 0.322879005 |
| ENSG00000110104 | 0.322845182 |
| ENSG00000126883 | 0.322643699 |
| ENSG00000119801 | 0.322198541 |
| ENSG00000164062 | 0.322152474 |
| ENSG00000130559 | 0.321755807 |
| ENSG00000099219 | 0.321639609 |
| ENSG00000226432 | 0.321342645 |
| ENSG00000175215 | 0.321136971 |
| ENSG00000107021 | 0.321111599 |
| ENSG00000023330 | 0.318388198 |
| ENSG00000086189 | 0.318137508 |
| ENSG00000135932 | 0.317456956 |
| ENSG00000167721 | 0.317116997 |

| | |
|---|---|
| ENSG00000082996 | 0.317112347 |
| ENSG00000125651 | 0.316485756 |
| ENSG00000164463 | 0.315559462 |
| ENSG00000112062 | 0.315505521 |
| ENSG00000198556 | 0.315477362 |
| ENSG00000148396 | 0.315040406 |
| ENSG00000150593 | 0.314782104 |
| ENSG00000107290 | 0.314646498 |
| ENSG00000148187 | 0.31410765 |
| ENSG00000213064 | 0.313609257 |
| ENSG00000096063 | 0.313250829 |
| ENSG00000177042 | 0.312209243 |
| ENSG00000088930 | 0.312084294 |
| ENSG00000148296 | 0.31107982 |
| ENSG00000110367 | 0.310794 |
| ENSG00000123992 | 0.310720153 |
| ENSG00000164091 | 0.31038497 |
| ENSG00000139842 | 0.310271374 |
| ENSG00000143321 | 0.310097123 |
| ENSG00000167747 | 0.309800917 |
| ENSG00000158122 | 0.309229613 |
| ENSG00000165283 | 0.308318218 |
| ENSG00000177666 | 0.308287415 |
| ENSG00000133398 | 0.308254743 |
| ENSG00000132823 | 0.307354978 |
| ENSG00000129292 | 0.307188001 |
| ENSG00000147996 | 0.306217548 |
| ENSG00000244005 | 0.305424103 |
| ENSG00000135968 | 0.305130515 |
| ENSG00000204160 | 0.304997352 |
| ENSG00000144746 | 0.304530864 |
| ENSG00000166986 | 0.304309099 |
| ENSG00000115365 | 0.303350313 |
| ENSG00000170903 | 0.303277805 |
| ENSG00000125826 | 0.302633939 |
| ENSG00000101343 | 0.301159742 |
| ENSG00000110906 | 0.30113318 |
| ENSG00000131323 | 0.300708349 |
| ENSG00000168259 | 0.300211479 |
| ENSG00000173875 | 0.299541038 |

| | |
|---|---|
| ENSG00000158552 | 0.299214343 |
| ENSG00000160563 | 0.297805962 |
| ENSG00000125734 | 0.297507911 |
| ENSG00000025293 | 0.297469494 |
| ENSG00000101474 | 0.297108237 |
| ENSG00000091039 | 0.296994512 |
| ENSG00000110911 | 0.296853818 |
| ENSG00000115685 | 0.296319002 |
| ENSG00000107164 | 0.295900484 |
| ENSG00000112624 | 0.294945233 |
| ENSG00000125484 | 0.294915513 |
| ENSG00000109572 | 0.294226873 |
| ENSG00000148291 | 0.292246716 |
| ENSG00000196227 | 0.291518755 |
| ENSG00000171940 | 0.290373833 |
| ENSG00000136816 | 0.289860561 |
| ENSG00000101391 | 0.288268467 |
| ENSG00000125871 | 0.288259381 |
| ENSG00000233137 | 0.28537084 |
| ENSG00000189180 | 0.281364366 |
| ENSG00000104218 | 0.275484847 |
| ENSG00000149474 | 0.274688018 |
| ENSG00000120686 | 0.274672684 |
| ENSG00000136003 | 0.273897222 |
| ENSG00000206560 | 0.273101118 |
| ENSG00000176101 | 0.27102652 |
